# Genomic analysis of oesophageal squamous-cell carcinoma identifies alcohol drinking-related mutation signature and genomic alterations

**DOI:** 10.1038/ncomms15290

**Published:** 2017-05-26

**Authors:** Jiang Chang, Wenle Tan, Zhiqiang Ling, Ruibin Xi, Mingming Shao, Mengjie Chen, Yingying Luo, Yanjie Zhao, Yun Liu, Xiancong Huang, Yuchao Xia, Jinlin Hu, Joel S. Parker, David Marron, Qionghua Cui, Linna Peng, Jiahui Chu, Hongmin Li, Zhongli Du, Yaling Han, Wen Tan, Zhihua Liu, Qimin Zhan, Yun Li, Weimin Mao, Chen Wu, Dongxin Lin

**Affiliations:** 1 Key Laboratory for Environment and Health (Ministry of Education), School of Public Health, Huazhong University of Science and Technology, No. 13 Hangkong Road, Wuhan 430030, China; 2 Department of Etiology and Carcinogenesis, National Cancer Center/Cancer Hospital, Chinese Academy of Medical Sciences and Peking Union Medical College, No.17 Panjiayuan Nanli, Chaoyang District, Beijing 100021, China; 3 Cancer Institute, Zhejiang Cancer Hospital, No. 38 Guangji Road, Hangzhou 310022, China; 4 Department of Probability and Statistics, School of Mathematical Sciences and Center for Statistical Science, Peking University, No. 5 Yiheyuan Road, Haidian District, Beijing 100871, China; 5 Department of Genetics, University of North Carolina, Chapel Hill, North Carolina 27599, USA; 6 Department of Biostatistics, University of North Carolina, Chapel Hill, North Carolina 27599, USA; 7 Department of Pathology, Zhejiang Cancer Hospital, No. 38 Guangji Road, Hangzhou 310022, China; 8 Department of Computer Science, University of North Carolina, Chapel Hill, North Carolina 27599, USA; 9 State Key Laboratory of Molecular Oncology, National Cancer Center/Cancer Hospital, Chinese Academy of Medical Sciences and Peking Union Medical College, No.17 Panjiayuan nanli, Chaoyang District, Beijing 100021, China; 10 Lineberger Comprehensive Cancer Center, University of North Carolina, Chapel Hill, North Carolina 27599, USA; 11 Department of Thoracic Surgery, Zhejiang Cancer Hospital, No. 38 Guangji Road, Hangzhou 310022, China

## Abstract

Approximately half of the world's 500,000 new oesophageal squamous-cell carcinoma (ESCC) cases each year occur in China. Here, we show whole-genome sequencing of DNA and RNA in 94 Chinese individuals with ESCC. We identify six mutational signatures (E1–E6), and Signature E4 is unique in ESCC linked to alcohol intake and genetic variants in alcohol-metabolizing enzymes. We discover significantly recurrent mutations in 20 protein-coding genes, 4 long non-coding RNAs and 10 untranslational regions. Functional analyses show six genes that have recurrent copy-number variants in three squamous-cell carcinomas (oesophageal, head and neck and lung) significantly promote cancer cell proliferation, migration and invasion. The most frequently affected genes by structural variation are *LRP1B* and *TTC28*. The aberrant cell cycle and PI3K-AKT pathways seem critical in ESCC. These results establish a comprehensive genomic landscape of ESCC and provide potential targets for precision treatment and prevention of the cancer.

## Abstract (web summary)

Oesophageal squamous-cell carcinoma (ESCC) is a leading cause of cancer death, and half of ESCC cases occur in China. Here, the authors provide an in depth genomic landscape for this disease and identify specific mutation signatures—one of which is linked to alcohol intake.

Oesophageal squamous-cell carcinoma (ESCC) ranks the fourth leading cause of cancer death and approximately half of the world's 500,000 new ESCC cases each year occur in China^1^,^2^. Epidemiological studies have suggested that alcohol intake, tobacco smoking, micronutrient deficiency and dietary carcinogen exposure may cause ESCC^3^,^4^,^5^. However, the mutational signatures in ESCC associated with lifestyle or environmental aetiological factors have not been explored. There are currently no specific molecule-targeting agents for ESCC treatment and therefore the long-term outcome of this cancer is still dismal, with 5-year survival rate around 30% (refs 6, 7).

Several studies on whole-exome sequencing (WES) in ESCC in Chinese and Japanese populations have been published recently. These studies reported an extremely high frequency of *TP53* mutations and low prevalence but statistically significant single-nucleotide variations (SNVs) in several other genes, including *CDKN2A*, *NOTCH1*, *RB1* and *PIK3CA*^8^,^9^,^10^,^11^,^12^,^13^,^14^,^15^. All these WES studies only took into account somatic variations in the protein-coding regions; however, the protein-coding components of the genome account for only ∼2% of the total sequences and previous studies indicated that the non-coding regions are more frequently affected by mutations compared with the coding regions^16^. The effects of the non-coding variations on ESCC development have not been characterized at the whole-genome level. Although both copy-number variations (CNVs) and structural variations (SVs) in ESCC have been reported, the results were based on low-coverage whole-genome sequencing (WGS) and/or single-nucleotide polymorphism (SNP) array with relatively small sample size^9^,^10^,^11^,^12^,^13^,^14^,^15^,^17^. The apparent limitations in the previous studies warrant performing more comprehensive studies.

Here we report an integrated analysis of genomic signatures, SNVs, CNVs, SVs and their correlations with mRNA expression in ESCC from Chinese individuals. We have comprehensively characterized the genomic landscape features in ESCC that may provide clues to environmental aetiological factors and identified potential targets that may guide to develop precision treatment and prevention of this malignancy.

## Results

### WGS of ESCC samples

We collected tumours, distant normal tissues, peripheral blood samples and clinical information from 94 individuals with ESCC (Supplementary Fig. 1 and Supplementary Data 1). WGS of the tumour and blood samples was performed and mutational signatures, SNVs, CNVs and SVs were analysed. In addition, we performed RNA sequencing of all ESCC and distant normal tissues. The mean coverage of WGS was 56 × for tumour with 94% of bases >30 × and 36 × for matched blood samples with 93% of bases >20 × , respectively (Supplementary Data 2). In all, WGS identified 617,629 SNVs, 55,453 indels, 57 focal CNV regions and 6,844 SVs (Supplementary Fig. 2a,b).

**Figure 1:**
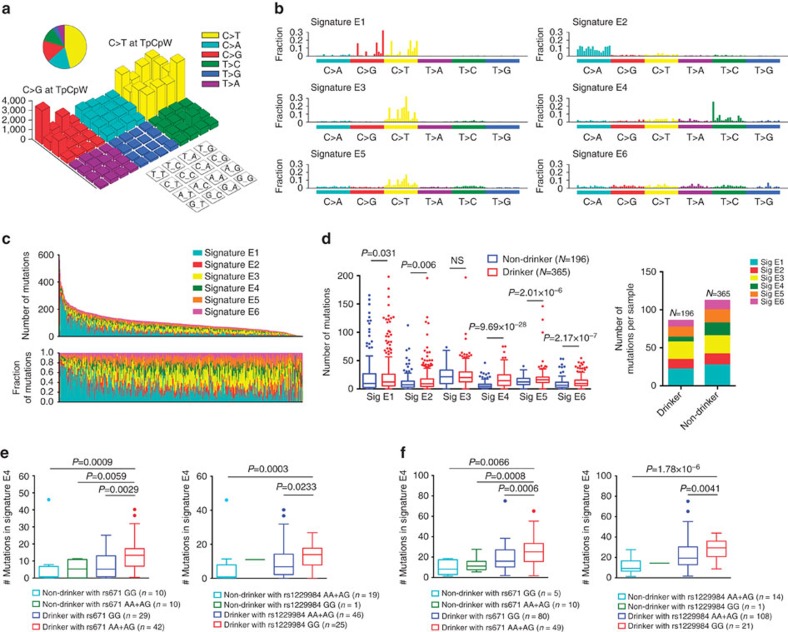
Genome-wide mutational signatures of 704 ESCC samples. (**a**) The whole-exome mutation spectra. Colours represent the six SNV types on the upper right. The three base content of each mutation is labelled in the 4 × 4 legend on the lower right. ESCC show an enrichment for APOBEC-mediated C>G and C>T mutations of C>G and C>T in TpCpW trinucleotide sites (where W corresponds to either A or T). (**b**) Patterns of substitutions for Signatures E1–E6. Each signature is displayed according to the 96 substitution classifications defined by the substitution class and sequence context immediately 5′ and 3′ to the mutated base. The vertical axis represents mutation fractions of each substitution classification. (**c**) The contributions of mutational signatures to individual ESCC samples. Each bar represents a selected sample from the 704 ESCC samples. The horizontal axis denotes 704 ESCC samples and the vertical axis denotes the number of mutations (upper panel) or mutation fractions (lower panel). (**d**) Number of mutations in each signature (left panel) and total number of mutation (right panel) as function of drinking status. The data represent median and interquartile range and *P* values are from unpaired Wilcoxon rank-sum test. NS, not significant. (**e**,**f**) Number of mutations in Signature E4 as function of *ALDH2* or *ADH1B* genotypes in Chinese (**e**) or Japanese (**f**) ESCC samples. Data are displayed in Tukey's boxplot. The line in the middle of the box is plotted at the median while the upper and lower hinges indicated 25th and 75th percentiles. Whiskers indicate 1.5 times interquartile range (IQR) and values greater than it are plotted as individual points. The minima and maxima are the lowest datum still within 1.5 IQR of the lower quartile and the highest datum still within 1.5 IQR of the upper quartile. Unpaired Wilcoxon rank-sum test were used. NS, not significant.

We found a genome-wide mutation rate of 2.1 mutations per megabase (Mb). There were 6,797 (1.1%) SNVs or indels with 1.98 mutations per Mb in the protein-coding regions and 610,832 (98.9%) SNVs or indels with 2.13 mutations per Mb in the non-coding regions, respectively (Supplementary Fig. 2a). The amount of SNVs was positively associated with mutational status of *TP53* (*P*=1.34 × 10^−4^, unpaired *t*-test; Supplementary Table 1). We found that 9.6% (9/94) of ESCC samples had occasional kataegis loci (range=1–3), which are localized substitution hypermutations and associated with genomic rearrangement, and 4 of these kataegis had SVs nearby (Supplementary Fig. 2c and Supplementary Table 2). We counted the number of SNVs in the matrix of 96 possible mutations occurring in a trinucleotide context in each ESCC sample and found that the predominant mutations were the C>T and C>G transitions at TpCpW trinucleotide sites (Fig. 1a), which is similar to the signature of APOBEC enzyme family-mediated mutagenesis^18^.

### The mutational signatures of ESCC

Mutational signatures in cancer genome might reflect and trace DNA damage caused by DNA-damaging agents to which cells have been exposed. However, the previous WES studies did not address this important issue in ESCC probably due to the limited statistical power. In this study, we combined our WGS data with WES data obtained from published studies^9^,^11^,^12^,^13^,^14^,^15^ including 94 ESCC samples from The Cancer Genome Atlas (TCGA) database to increase sample size to 704 for analysing SNV profile in the protein-coding regions. By using 1,000 iterations of non-negative matrix factorization^19^, we identified six mutational signatures (Signatures E1–E6) in ESCC that are of high stability and low reconstruction error (Supplementary Fig. 3).

We then performed cosine similarity analysis to compare the mutational signatures in ESCC with current Catalogue of Somatic Mutations in Cancer (COSMIC)^19^ (Supplementary Table 3) and found that five ESCC signatures are highly similar to COSMIC signatures. Signature E1 is highly similar to COSMIC Signatures 2 and 13 (cosine similarity 0.819 and 0.813, respectively), which is believed to be due to over activity of the APOBEC, a family of cytidine deaminases. APOBEC enzymes convert cytidine to uracil that is usually coupled with the activities of base excision repair and DNA replication machineries^20^. In our samples, the expression levels of *APOBEC3B* and *APOBEC3C* were significantly higher in tumours than in paired normal tissues (*P*=8.81 × 10^−31^ and *P*=3.21 × 10^−5^, respectively, unpaired *t*-test; Supplementary Fig. 2d) and strongly correlated with the total number of the C>T (*P=*0.022, Spearman's *r*=0.24) and C>G (*P=*0.019, Spearman's *r*=0.24) mutations at TpCpW trinucleotide sites, respectively (Supplementary Fig. 2d). Signature E2 showed high similarity to COSMIC Signature 4 (cosine similarity 0.860), which was previously identified to be associated with smoking status in lung cancer patients^19^. This signature is only significantly associated with individuals' smoking status in our 94 ESCC samples (*P*=0.027, unpaired Wilcoxon rank-sum test; Supplementary Fig. 4c) but not in other WES studies (Supplementary Fig. 4b,c). Signature E3 (cosine similarity 0.829 and 0.783) and Signature E5 (cosine similarity 0.838 and 0.892) are highly similar to COSMIC Signatures 1A and 1B, both of which were identified as age-dependent mutational signatures (Supplementary Table 3). Signature E3 and Signature E5 were both associated with age at diagnosis of 699 ESCC who had information on age (*P*=3.20 × 10^−4^ and *P*=0.006, unpaired Wilcoxon rank-sum test; Supplementary Fig. 4a). We found that the average percentage of the T>C mutation per sample was significantly higher in drinkers (13.6%) than in non-drinkers (9.7%; *P*=3.60 × 10^−10^, unpaired *t*-test) and Signature E4, specifically characterized by the T>C mutation, is similar to COSMIC Signature 16 (cosine similarity 0.873) of which the origin was unknown before. In the present study, we identified Signature E4 significantly associated with individuals' smoking and drinking status (*P*=0.002 and *P*=9.69 × 10^−28^, unpaired Wilcoxon rank-sum test; Fig. 1d and Supplementary Fig. 4b). Our previous genome-wide association studies have identified two functional SNPs, rs671 in *ALDH2* on 4q23 and rs1229984 in *ADH1B* on 12q24, that are significantly associated with the risk of ESCC in a manner of interactions with alcohol drinking and tobacco smoking status^21^,^22^. Therefore, we further analysed the mutation profiles in the function of *ALDH2* and *ADH1B* genotypes. We found that the frequency of Signature E4 mutations in ESCC from drinkers with the risk *ALDH2* genotype (rs671*-AG/-AA*) was significantly higher than that in ESCC from drinkers with the non-risk genotype (rs671*-GG*; *P*=0.003, unpaired Wilcoxon rank-sum test). It was also significantly higher than that in ESCC from non-drinkers with the rs671*-AG/-AA* genotype (*P*=0.006, unpaired Wilcoxon rank-sum test) or ESCC from non-drinkers with the rs671*-GG* genotype (*P*=0.001, unpaired Wilcoxon rank-sum test) (Fig. 1e). The significant differences between the frequencies of Signature E4 mutations were also seen when the analysis was stratified by the *ADH1B* rs1229984 genotypes (Fig. 1e). Interestingly, the similar results were also observed in ESCC in Japanese^14^, whose *ALDH2* and *ADH1B* genotype data are available (Fig. 1f). We also found that individuals with Signature E4 mutations had significant transcriptional strand bias for the T>C change (Supplementary Fig. 5), which is believed to be caused by the differences in repair efficiency of DNA damage and maintenance processes between transcribed and untranscribed strands of genes^19^. Signature E6 (Fig. 1b), characterized specifically by the T>A and T>G mutations, showed low similarity to any of the COSMIC signatures (all cosine similarity <0.8) and appears to be a new mutational signature in ESCC.

### Recurrent mutations in the protein-coding genes

We obtained 6,184 SNVs and 613 indels in the exonic regions of 94 ESCC samples (Supplementary Data 3); 72% of these mutations were expressed and 41, 46 and 53% mutations were confirmed if the locus has at least 10, 5 and 1 read/s, respectively, with the mutant nucleotide by RNA sequencing. In addition, we selected 105 putative somatic mutations for further validation and 95 (90.5%) selected mutations were confirmed by PCR-based Sanger sequencing. Using the MutSigCV^23^, we identified six significantly mutated genes, including *CDKN2A*, *TP53*, *NOTCH1*, *FBXW7*, *NFE2L2* and *AJUBA* (all false discovery rate (FDR) *q*<0.5; Supplementary Fig. 6). All of them have previously been reported in ESCC and two other types of squamous cell carcinomas, head and neck squamous cell carcinomas (HNSCC) and lung squamous cell carcinomas (LUSCC) (Supplementary Data 4).

**Figure 2:**
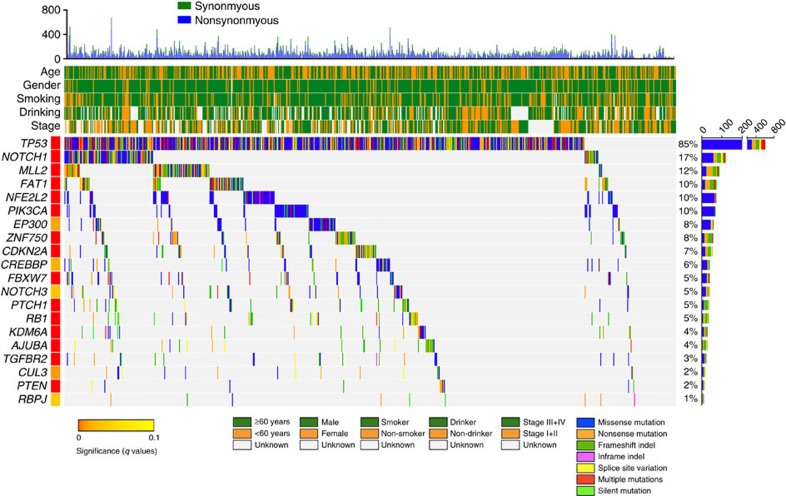
Mutational landscape of somatic alterations in 704 ESCC samples. Significantly mutated genes (identified using the MutSigCV algorithm; FDR *q*<0.1) are ordered by *q* value. Samples are arranged to emphasize mutual exclusivity among mutations. Each column denotes an individual tumour, and each row represents a gene. Very top, total number of mutations (*y* axis) for each sample (*x* axis). Top, key clinical parameters of each examined case. Right, percentage of mutation in 704 ESCC samples while the vertical axis represents total number of mutations for each gene. Clinical characteristics and mutation types are shown by colour as indicated.

To increase the statistical power to detect the driver mutations, we analysed the data in combined sample of 704 ESCC, which provides 90% power to detect genes mutated in 2% individuals^24^. As a result, we detected 20 putative driver genes and found that 93% (657/704) of ESCC had at least one somatic mutation in these genes (Fig. 2 and Supplementary Figs 7 and 8). Recapitulating previous reports, we confirmed mutations in 12 genes, including *MLL2*, *FAT1*, *PIK3CA*, *EP300*, *ZNF750*, *CREBBP*, *NOTCH3*, *PTCH1*, *RB1*, *KDM6A*, *TGFBR2* and *PTEN*. Furthermore, we identified two novel mutated genes, *CUL3* and *RBPJ*, in ESCC (Fig. 2 and Supplementary Data 4). We found that knockdown of *CUL3* or *RBPJ* significantly promoted malignant phenotypes of ESCC cells such as proliferation, migration and invasion; but overexpression of these genes significantly suppressed these malignant phenotypes (Supplementary Fig. 9). In addition, we observed that the rate of *PTCH1* mutation was significantly higher in ESCC in smokers compared with that in nonsmokers (*P*=0.030, one-sided Fisher's exact test), while the rates of *TP53*, *EP300*, *PTCH1*, *NOTCH3*, *TGFBR2* and *ZNF750* mutations were significantly higher in ESCC in drinkers than that in non-drinkers (all *P*<0.05, one-sided Fisher's exact test). ESCC at late stage had significantly more *NOTCH1* mutations compared with ESCC patients at early stage (*P*=0.001, one-sided Fisher's exact test; Supplementary Data 5).

We then compared the mutational profiles in ESCC, HNSCC, LUSCC or oesophageal adenocarcinoma (EAC) and found that the 20 genes significantly mutated in ESCC were also frequently mutated in HNSCC and LUSCC; however, in EAC, only *TP53*, *CDKN2A* and *PIK3CA* were frequently mutated (Supplementary Data 4). Clustering analysis showed that the mutational profile of ESCC is similar to HNSCC and LUSCC but dissimilar to EAC (Supplementary Fig. 10). These results indicate that cancers originated from the same cell type might have the same driver mutations but cancers in the same organ generated from different cell type might have distinct driver mutations, reflecting different aetiology and the molecular mechanisms of pathogenesis^15^,^25^.

### Recurrent mutations in the non-coding elements

We next analysed the mutations in the non-coding regions, including the gene promoters, untranslated regions (UTRs) and long non-coding RNAs (lncRNAs) annotated by GENCODE v24 (ref. 26). Using the regional recurrence testing approach^16^, we found 15 regions that had significantly higher mutation rates than that expected by chance (*q*<0.05; Supplementary Table 4 and Supplementary Fig. 11), including 4 lncRNAs (*NEAT1*, *LINC01360*, *PART1* and *CTA-280A3.2*), 1 promoter (*FCMR*) and 10 UTRs (*SMAD2*, *YIPF4*, *ZNF605*, *FOXJ3*, *NABP1*, *CLOCK*, *YTHDC1*, *TMEM178B*, *TNFRSF11A* and *GALR1*). For instance, 6.4% ESCC samples had mutations in *NEAT1*, an lncRNA that is associated with malignant phenotypes of several types of cancer including ESCC^27^,^28^,^29^,^30^. By integrating analysis of WGS and RNA sequencing data, we found that the mutations in the 3′UTR of *FOXJ3* were significantly correlated with the overexpression of this gene, whereas the mutations in the 3′UTR of *CLOCK* were significantly associated with downregulation of this gene (Supplementary Fig. 12).

### Whole-genome copy-number alterations

**Figure 3:**
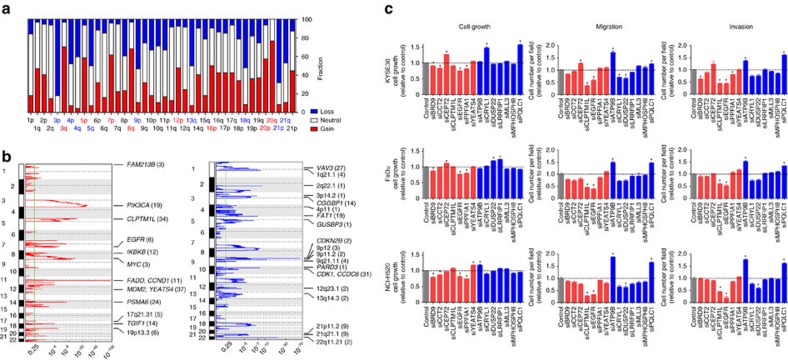
CNV in ESCC and functional impact of some genes with CNV. (**a**) CNV at arm level. The bar graphs show the frequency of arm-level copy-number alterations and the vertical axis denotes chromosome arms. (**b**) CNV at focal regions detected by GISTIC 2.0. Regions of recurrent focal amplifications (left) and focal deletions (right) are plotted by false discovery rate (*x* axis) for each chromosome (*y* axis). Annotated peaks have residual *q*<0.25 and ≤40 genes within peak regions. These peak regions are annotated with candidate known cancer genes and the total number of genes within these peaks are given in brackets. A dashed line represents the centromere of each chromosome. (**c**) The effects of knockdown by siRNA of 14 genes with CNV on proliferation, migration and invasion of SCC cell lines KYSE30, FaDu and NCI-H520. These genes were selected for functional assays because they were affected by CNV identified in all three types of SCC, that is, ESCC, HNSCC and LUSCC. For cell proliferation, the results are presented as mean±s.e.m. from three independent experiments and each had three replications. For cell migration and invasion, the results are presented as mean±s.e.m. from three independent experiments and each had duplication. The dashed line represents mean of siRNA control result. *P<0.01 compared with control by Student's *t*-test.

We found that 97.9% (92/94) of ESCC had CNVs at chromosome arm level, including loss at 3p, 4p, 4q, 5q, 9p, 13q, 18q and 21p and gain at 3q, 5p, 7p, 8q, 12p, 16p, 20p and 20q (Fig. 3a). These CNV profiles resemble those in HNSCC and LUSCC but differ from those in EAC and stomach adenocarcinoma (STAD, Supplementary Fig. 13a,b). Through additional cluster analysis of RNA expression data, we found that the RNA expression pattern in ESCC is also similar to that in HNSCC and LUSCC but differs from that in EAC and STAD (Supplementary Fig. 13c,d). The similar SNV and CNV profiles along with the similar RNA expression pattern support that ESCC belongs to the ‘squamous' molecular subtype as identified by Hoadley *et al*.^31^ We also identified 23 focal regions with recurrent gain of copy number and 34 regions with recurrent loss of copy number (both *q*<0.25; Fig. 3b), in which 1,591 and 4,841 genes are involved, respectively, and many have previously been identified as tumour-associated genes (Supplementary Data 6 and 7). Among them, five recurrent focal amplifications (1p36.32, 1q42.13, 6p21.33, 17q21.31 and 19p13.3) and seven recurrent focal deletions (5q13.2, 6p24.3, 10q21.1, 12q23.1, 13q31.3, 15q14 and 21q21.1) were identified for the first time in ESCC (Supplementary Data 8).

We then retrieved these 57 focal CNV peaks and examined whether the gene with copy-number gain or loss has substantial effect on its mRNA expression measured by RNA sequencing. The results showed that, among the 6,432 genes located in these CNV regions, 538 (33.8%) with copy-number gain and 1,416 (29.3%) with copy-number loss had mRNA levels that are significantly correlated with gene copy number (Spearman's *r*>0.3 and *P*<0.05; Supplementary Fig. 14 and Supplementary Data 9). Through the analysis using a gene–drug interaction database^32^, we found that, among the genes that show aberrations in DNA copy number and mRNA expression, 184 (including *EGFR*, *CCND1*, *PIK3CA* and *MAPK1*) have the potential to interact with at least one drug (Supplementary Data 10).

### Functional characterization of genes with CNV

To identify the causal copy-number alterations, we compared the CNV profile in ESCC with that in HNSCC and LUSCC. We found that 14 genes (7 with copy-number gain and 7 with copy-number loss) had aberrant mRNA expression in all three SCCs (Spearman's *r*>0.3 and *P*<0.05; Supplementary Data 11, Supplementary Figs 15–17). We then used a quick *in vitro* function screening approach to examine whether these 14 genes have effects on malignant phenotypes of SCC cell lines, KYSE30 (ESCC), DaFu (HNSCC) and NCI-H520 (LUSCC) and found that knockdown of *EGFR*, *BRD9* or *PPFIA1* expression by short interfering RNA (siRNA) resulted in significant inhibition of cell proliferation (Fig. 3c and Supplementary Figs 18–20). In addition, we found that knockdown of *EGFR* and *CLPTM1L* expression significantly inhibited migration and invasion, whereas knockdown of *ATP9B* or *PQLC1* significantly enhanced migration and invasion of cancer cells (Fig. 3c and Supplementary Figs 18–20).

### Whole-genome structural variations

**Figure 4:**
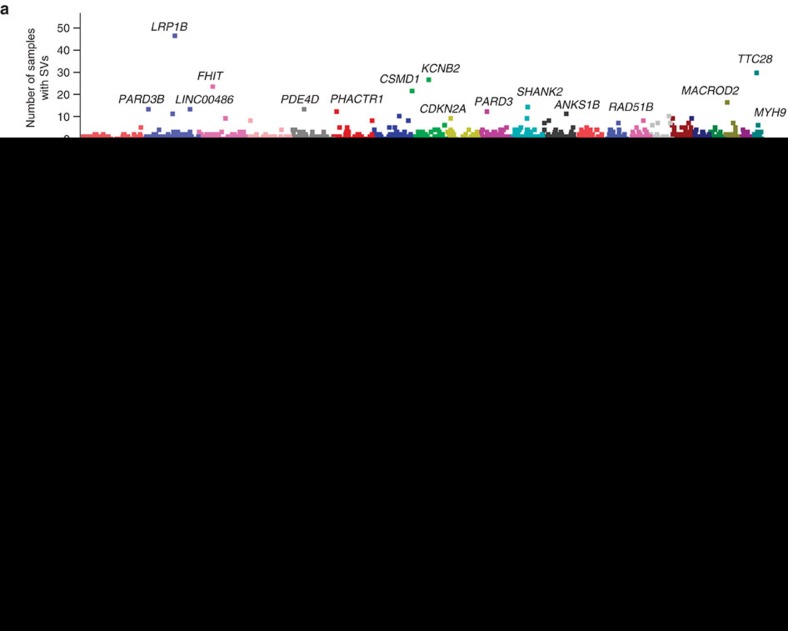
SVs and their effects on gene expression. (**a**) Number of samples with at least one SV breakpoint in gene region. The vertical axis represents chromosome positions. (**b**) Correlations of SV with mRNA expression of genes. The *y* axis denotes log-transformed *P* values from *t*-test between gene expression of samples with and without SV. The vertical axis represents chromosome positions. (**c**) Validation of gene fusion in ESCC. Left, gel analysis of PCR product of DNA from tumour and normal tissues; Right, Sanger sequencing of the amplicon.

We found 6,844 SVs in 94 ESCC samples (73 SVs per sample in average), including 3,610 (52.7%) inter-chromosomal translocations, 1,863 (27.2%) deletions, 805 (11.8%) inversions and 566 (8.3%) intra-chromosomal translocations. We also identified 13 (13.8%) chromothripsis (Supplementary Fig. 21), the massive DNA rearrangements resulting from the repair of localized chromosome shatters in a one-off catastrophe^33^,^34^. Across whole genomes of 94 ESCC, 2,607 SVs with breakpoints in 257 gene regions were found in >3 samples. The most frequently affected genes were *LRP1B* (48.9%), a putative tumour suppressor that displays frequent deletion or methylation in human cancer^35^,^36^ and *TTC28* (31.9%), whose recurrent chromosomal translocation was also seen in colorectal cancer, small-cell lung cancer and liver cancer^28^,^37^,^38^ (Fig. 4a). We also identified SVs in *FHIT*, *RAD51B*, *MYH9* and *CDKN2A* in a frequency of >5% samples (Supplementary Data 12). Since SVs may alter gene expression due to the change of gene-enhancer distance or alterations of gene copy number or structure, we therefore examined the mRNA expression of genes with or without SVs in ESCC (Fig. 4b). Interestingly, we found 23 genes whose expression were significantly altered by SV (*P*<0.05, unpaired *t*-test; Supplementary Data 12) and among them were well-known cancer-related genes *CDKN2A* (SV in 5 samples), *PRKCA* (SV in 3 samples) and *EP300* (SV in 3 samples).

We further screened gene fusion events in our samples, which were validated by PCR-based Sanger sequencing (Supplementary Table 5). We identified *ERC1* that was fused with different partners, *PRAF2*, *WNK1* or *RAD51*, respectively, in three samples. *ERC1* is a member of RIM-binding protein family that regulates neurotransmitter release and has been shown to be fused to the *RET* in papillary thyroid carcinoma^39^. We also detected and validated *NRG1-ZCCHC7* and *WT1-MRPL19* fusions, respectively, in two samples (Fig. 4c).

### Functionally aberrant pathways in ESCC

**Figure 5:**
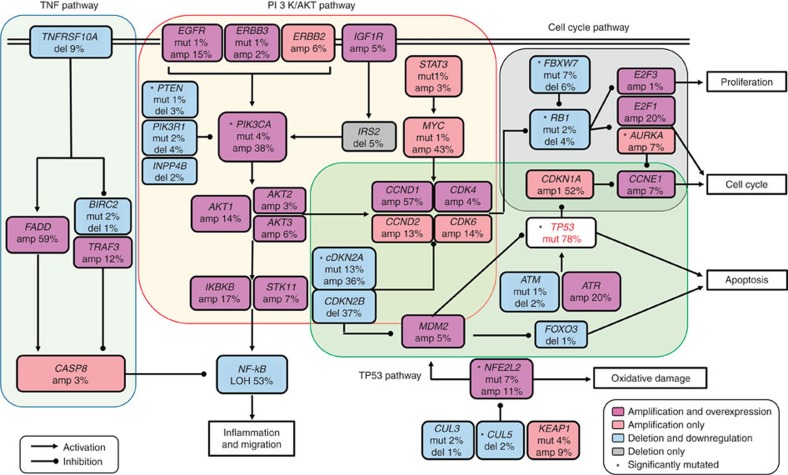
Significantly aberrant pathways and networks in ESCC. The rectangles in different colours represents percentages of amplification, amplification and overexpression, deletion, deletion and low expression and mutations in genes identified in ESCC that belong to four signaling pathways as indicated in the indicators. The amplification and deletion were defined by CNV analysis (all_thresholded.by_genes.txt file from the GISTIC output). The overexpression or low expression were defined by paired *t*-test between tumour and normal tissues with *P*<0.05 and sexpression fold change >1.2 or <0.8 being considered to be significant.

We integrated the above described 20 genes having driver mutations and 586 genes with CNV into pathway analysis to identify the functionally aberrant pathways in ESCC. We selected the genes with CNV for the analysis based on both copy number and mRNA expression changes in tumour tissues compared with normal tissues. As a result, we identified a highly interconnected network of aberrations targeting the receptor tyrosine kinases (RTK/RAS/phosphoinositide-3 kinase (PI3K)), cell cycle regulators, WNT, NOTCH and TP53 pathways (Fig. 5, Supplementary Data 13 and 14). Several receptor tyrosine kinases (that is, *EGFR*, *ERBB2* and *ERBB3*), their downstream signal transducer *PIK3CA* and other components in the RTK/PI3K pathway exhibited recurrent focal amplification and/or SNV. We found 14.9% (14/94) samples that had *EGFR* amplification while SNV in this gene was only 1.1% (1/94). The amplification rate for *PIK3CA*, a known oncogene in many types of human cancer, was 38.3% (36/94) and only two samples with *PIK3CA* amplification displayed concurrent mutations. We found a significant positive correlation between *PIK3CA* copy-number gain and elevated mRNA expression. The deletion rate of *PIK3R1* in our samples was 4.3% (4/94) and the mRNA expression levels of this gene were significantly lower in tumours than in normal tissues (*P=*3.71 × 10^−24^, paired *t*-test).

We found that several genes involved in the cell cycle pathway (for example, *TP53*, *CDKN2A*, *CCND1* and *MYC*) were frequently altered. *P53* was mutated in 77.7% (73/94) of samples, consistent with the previous reports^8^,^9^,^10^,^11^,^12^,^13^,^14^,^15^, while *CDKN2A* was deleted or mutated in 36.2% (34/94) or 12.8% (12/94) of samples. *CCND1* and *MYC* were frequently amplified with a rate of 57.4% (54/94) and 42.6% (40/94). Furthermore, we found *RB1* mutation in 2.1% (2/94) and deletion in 4.3% (4/94) of samples. Loss of *CDKN2A* and/or gain of *CCND1* may impair RB-mediated cell cycle control^40^. *CDKN2A* is a tumour suppressor in the development of squamous cell carcinoma and other types of human cancer^41^,^42^. Cyclin D1 encoded by *CCND1* can form a complex with CDK4 and CDK6, which may lead to RB protein phosphorylation, resulting in disruption of cell cycle restriction, genome instability and tumorigenesis^43^.

In addition, we identified mutation and amplification of *NFE2L2* (also known as *NRF2*), a key transcriptional regulator involved in metabolic processes and oxidative stress^44^, with a rate of 7.4% (7/94) and 10.6% (10/94). We also identified *CUL3* mutation and deletion in 2.1% (2/94) and 1.1% (1/94), respectively, and *CUL5* deletion in 2.1% (2/94) of samples, both of which encode proteins that form a complex with NFE2L2 in transcriptional regulation. Mutations in *NFE2L2* may result in increased activity of its protein and consequent deregulation of its downstream molecules that promote tumorigenic metabolism. We also identified mutations in *NOTCH* gene family, which play important roles in SCCs^45^,^46^,^47^. We found significantly elevated mutations in *NOTCH1* (17.0% (16/94)) and *NOTCH3* (5.3% (5/94)) and non-significant (*q*>0.1) mutations in *NOTCH2* (3.2% (3/94)). Two genes encoding the epigenetic regulators, *MLL2* and *CREBBP*, were mutated in 13.8% (13/94) and 5.3% (5/94) of samples and its amplification occurred with a frequency of 1.1% (1/94) and 5.3% (5/94). *FBXW7*, a well-established tumour-suppressor gene that targets various oncogenic proteins for degradation, was mutated in 7.4% (7/94) and deleted in 6.4% (6/94) of samples.

## Discussion

Several studies on whole-exon sequencing of ESCC have recently been published; however, the results are limited to somatic mutations in exonic regions of protein-coding genes, the approximately 2% of DNA sequences in human genome. In the present study, we performed an integrative analysis of WGS and RNA sequencing of 94 ESCC samples. Based on the systematical analysis of whole-genome mutational signatures, SNVs, CNVs, SVs and mRNA expression and combined analysis with previous WES data, we have established a more comprehensive genomic landscape of ESCC. Several novel findings have been achieved in this study. First, we identified six mutational signatures in ESCC and found that Signature E4, characterized by the T>C mutation in the transcribed strand, is associated with alcohol intake and germline variants in two alcohol-metabolizing genes. Second, we identified recurrent mutations in 20 protein-coding genes, among them were *CUL3* and *RBPJ*, two genes whose recurrent mutations have never been reported before in ESCC. We also identified for the first time the significant mutations in 15 non-coding regions, including that produces lncRNA *NEAT1* (refs 27, 28, 29, 30) and 3′UTR of *FOXJ3* and *CLOCK*. Third, we identified 57 focal regions with recurrent CNVs and 6 genes in these regions had aberrant mRNA expression, which significantly promoted proliferation, migration and/or invasion of SCC cells. Furthermore, we identified that *LRP1B* and *TTC28* are the most frequently affected genes by SV in ESCC. We also found frequent aberrations of cell cycle pathway and PI3K-AKT signaling pathway in ESCC, suggesting that these may be the most critical pathways involved in the development and progression of ESCC.

In the present study, we combined available ESCC WES data with our WGS results (total *N*=704) for analysis of mutational signatures and SNVs in coding regions^9^,^11^,^12^,^13^,^14^,^15^. We did not include the first whole-exonic sequencing study^8^ for combined analysis because of small sample size of only 12 ESCC. Using combined analysis with increased sample size, we not only confirmed 18 significant SNVs but also identified two novel recurrent mutations in the *CUL3* and *RBPJ* genes. *CUL3* encodes cullin 3, a member of a ubiquitin ligase complex that functions in the oxidative stress response pathway. This gene displays frequent deletions or mutations in SCC^47^,^48^. *RBPJ* encodes a transcriptional factor regulating Notch signaling pathway, which has been shown to be an effective therapeutic target in various tumours^45^,^49^,^50^. By using this large sample size, we generated clinical background-related mutational signatures in ESCC. More importantly, we found that, among the six mutational signatures, Signature E4 occurred mostly in the transcribed DNA strand and was correlated with patients' drinking status and the variant genotypes in *ALDH2* and *ADH1B* that have been associated with increased susceptibility to ESCC by genome-wide association studies^21^,^22^. The correlations between mutations in Signature E4 in ESCC and drinking and alcohol-metabolizing genotypes further support an important role of alcohol intake in aetiology of ESCC^4^,^5^.

In this study, we also performed functional assays to validate the novel findings. We found that disturbing *CUL3* and *RBPJ* expression substantially altered the malignant phenotypes, such as proliferation, migration and invasion *in vitro* in SCC cells. Therefore, it is biologically plausible to define these two mutations as potential driver mutations in ESCC. We also demonstrated that knockdown of *EGFR*, *BRD9* and *PPFIA1*, which showed frequently copy-number gain at the genomic level, significantly inhibited cancer cell proliferation, indicating that these genes may play important oncogenic roles in ESCC. Moreover, we found that knockdown of expression of *CLPTM1L*, *ATP9B*, *PQLC1* as well as *EGFR* significantly inhibited cancer cell migration and invasion. While the effect of *CLPTM1L* on the progression of cancer has been previously reported^51^, the functions of *ATP9B* and *PQLC1* in SCC cell migration and invasion have never been reported before. These findings provide evidence that gain of function of these genes in ESCC might serve as therapeutic targets for ESCC treatment.

Non-coding DNA elements comprise the majority of the genome, and mutations in these regions have not been well characterized in ESCC. Accumulating evidence has indicated that lncRNAs play important roles in cancer^52^. Mutations in lncRNAs might cause RNA structure change or influence binding affinity to proteins. It has been shown that *NEAT1*, which forms SWI/SNF chromatin-remodelling complex^53^, participates in carcinogenesis of various types of cancer^27^,^28^,^29^,^30^, consistent with our finding that *NEAT1* is significantly mutated in ESCC. On the other hand, mutations in gene regulatory regions might change the binding affinity of miRNAs or transcriptional factors and result in alterations of gene expression. In this study, we indeed found that the mRNA expression levels in samples with mutations in 3′UTR of *FOXJ3* and *CLOCK* had significantly altered compared with samples without the mutations. *FOXJ3* and *CLOCK* have been reported to be involved in tumorigenesis^54^,^55^, which is in line with our findings. However, due to relative small sample size and low coverage of WGS in this study, further validation of the identified mutations in non-coding regions is needed.

Our present study has some limitations. First, the functional roles of the four mutated noncoding RNAs and their mutations are not clear yet and need to be addressed by further functional studies. Second, we identified several genes (for example, *LRPIB*, *FHIT* and *TTC28*) with high frequency of SV but the expression of these genes was not significantly changed. Although this phenomenon has also been observed in the WGS study of hepatocellular carcinoma and other WES studies^28^,^38^,^56^, further studies are warranted to elucidate the potential reasons, such as whether the SV occurs only in one allele but the gene expression is complemented by the other allele, whether there exist other mechanism such as SNV, CNV or DNA methylation, all of which may influence the expression of the affected genes, and whether these SVs may occur only in part of the whole gene region leading to a change of structure but not the expression of the gene.

In conclusion, this study characterized the genomic alterations in ESCC through WGS and integrative analysis. We have not only confirmed some previously reported findings but also identified several novel genomic alterations in ESCC. These results establish a comprehensive genomic landscape of ESCC and provide potential targets for precision treatment and prevention of the cancer.

## Methods

### Collection of biospecimens

ESCC were obtained from individuals (*N*=94) recruited at the Cancer Hospital, Chinese Academy of Medical Sciences (Beijing, China) and Zhejiang Cancer Hospital (Hangzhou, China) between 2010 and 2014. All the individuals with ESCC were unrelated Han Chinese whose demography characteristics and clinical information were obtained from medical records. Informed consent was obtained from each participant, and this study was approved by the Institutional Review Board of the Chinese Academy of Medical Sciences, Cancer Institute and Zhejiang Cancer Hospital. All the individuals underwent oesophagectomy and received no chemotherapy or radiotherapy before surgery. The biospecimens, including tumour tissues, adjacent normal oesophageal tissues (≥5 cm from tumour site) and blood samples from each individual, were collected at the time of treatment.

### Collection of clinical data

Clinical data for each individual were collected, which included gender, age at diagnosis, ethnicity, smoking status, drinking status, tumour site in the oesophagus, tumour stage and tumour grade. Individuals who smoked an average of <1 cigarette per day and for <1 year in their lifetime were defined as nonsmokers; otherwise, they were defined as smokers. Individuals were classified as drinkers if they drank at least twice a week and continuously for at least 1 year during their lifetime; otherwise, they were defined as nondrinkers. Tumour TNM staging components including Tumour, Node and Metastasis were reviewed by at least three pathologists and defined according to the American Joint Committee on Cancer Seventh edition. Previous studies have investigated numerous molecular and clinical risk factors without validation for potential clinical applications. Therefore, few patients had documentation for any molecular biomarkers for further analyses. Distributions of selected characteristics among the 94 patients are shown in Supplementary Table 6.

### Verification of histopathologically diagnosis

All the tumours were histopathologically classified as ESCC. Tumour samples and distant normal tissues were embedded in optimal cutting temperature medium and histological sections stained with haematoxylin and eosin were reviewed by at least two pathologists for quality assurance that tumour specimens contained an average of 75% tumour cell nuclei with <20% necrosis, whereas normal specimens contained no tumour cells.

### Processing of biospecimens

DNA and RNA from tissue samples were extracted using the Allprep DNA/RNA Kit (Qiagen) while blood DNA was extracted using QiaAmp Blood Midi Kit (Qiagen). DNA samples were quantified by Picogreen fluorescence assay and resolved by 1% agarose gel electrophoresis to confirm high molecular weight fragments. A custom Sequenom SNP panel was utilized to verify that tumour DNA and germline DNA was derived from the same patient. One microgram of DNA from each tumour and normal tissue was sent to Qiagen for REPLI-g whole-genome amplification using a 100 μg reaction scale. RNA was quantified by Abs260 using an UV spectrophotometer and then analysed via the RNA6000 nano assay (Agilent) for determination of an RNA Integrity Number. We discarded samples with RNA Integrity Number <7.0 (Supplementary Fig. 1). Aliquots of DNA and RNA were shipped to the sequencing centre for all subsequent testing.

### Genomic mutation detection and analysis

High-coverage WGS sequencing of DNA samples from 94 ESCC was performed on the Illumina HiSeq X Ten System. The sequence reads were aligned to the genome (hg19) using the *bwa* mem (v0.7.4)^57^ with default parameters. Duplicates were removed using Picard. The somatic mutations were called by Strelka^58^. Final somatic calls were filtered to require a quality score of >30 and were further annotated by snpEff and snpSift^59^. Non-negative matrix factorization was applied to the 96-substitution pattern and the contribution of each signature to each mutation catalogue was estimated by an algorithm from the Wellcome Trust Sanger Institute^19^. We performed PCR-based Sanger sequencing on a total of 105 putative somatic mutations identified in this study. PCR primers were designed using Primer3 to amplify a 100- to 700-bp region containing each mutation (Supplementary Data 15).

We carried out significance analysis of mutations using the MutSigCV algorithm^22^, as elaborated in several genome projects, with *q*<0.5 being considered significant in our data and *q*<0.1 being considered significant in the combined analysis. Genes mutated in <1% of samples or expressed (RSEM value of RNA sequencing is not zero) in <10% of samples were considered non-ESCC driver genes. For mutations in the non-coding regions, we performed analysis on promoters and UTRs of coding genes (*N*=20,014) and lncRNAs (*N*=7,747) annotated by Gencode v24 (ref. 26). The promoter is defined as the region between upstream 2 kb and downstream 250 bp from any transcription start site of a coding transcript gene and excluded any overlap with the coding regions. A regional recurrence testing approach was used to search for significant regions that were mutated more frequently than expected by chance^16^. To avoid false positive, only regions with FDR *q*<0.05 and >3% mutation samples were considered significant. Mutual exclusive analysis was performed using gitools (v2.2.2)^60^. All statistical analyses were performed in R using standard implementation.

We analysed copy number using SynthEx (unpublished, http://chenmengjie.github.io/SynthEx). In brief, 100 kb bin counts data (segment data) were generated using BEDTools^61^. The read ratios were calculated using the ‘synthetic normal' strategy described in SynthEx by averaging normal samples sequenced by the same protocol to reduce unwanted variation contributed by local characteristics and factors in sequencing process. A trending filter procedure was applied to segment the genome. The log2 ratios for segments were finally corrected by purity estimate from SynthEx. To infer recurrently focal peak in amplified or deleted genomic regions, we used the GISTIC algorithm^62^ with copy numbers in 1 kb windows as markers instead of SNP array probes. *G*-scores were calculated for genomic and gene-coding regions based on the frequency and amplitude of each gene with amplification or deletion. A significant CNV region was defined as having amplification or deletion with an FDR *q* value <0.25. For the arm-level analysis, chromosomal arms were considered to be altered if at least 66% of the arm (all_thresholded.by_genes.txt file from the GISTIC output) was lost or gained.

We used *Meerkat*^63^ and *Delly2* (ref. 64) to detect somatic SVs. Since the mean insert size is <300 bp and our WGS data are 150 bp paired-end reads, we first truncated the reads to 100 bp to avoid overlapping of the read pairs. Possible false positives were then filtered using a realignment procedure based on a hidden Markov Model and realigns discordant reads against a set of candidate sequences, including the local reference sequence and sequences obtained by inserting SNPs and indels implied in the short reads mapped nearby^65^. We then merged *Meerkat* and *Delly2* predictions: if the breakpoints of a SV are within 500 bp of those of another SV, the two SVs are merged and viewed as one single SV. Complex rearrangements are called by *ShatterSeek*^66^ and a genome having complex rearrangement is called if *P* value <0.01. All genes were annotated and enriched with Kyoto Encyclopedia of Genes and Genomes pathway using DAVID (v6.8)^67^.

### RNA expression analysis

Methods for RNA sequencing and data processing were based on the TCGA RNA-Seq protocol^38^. Briefly, total RNA of each sample was extracted, prepared into Illumina TruSeq mRNA libraries and sequenced by Illumina HiSeq2000 with a total of 10 Gb data. RNA reads were aligned to the hg19 genome assembly using Mapsplice^68^. To confirm SNVs reported in the WGS DNA samples, we applied UNCeqR^69^ to call somatic variants from RNA-Seq data. Gene expression using RSEM^47^ was quantified for the transcript models corresponding to the TCGA GAF2.1 and normalized to a fixed upper quartile of total reads within the sample. For further gene-level analysis, expression values were the RSEM value added by 1 and then log2 transformed. Principal component analysis (PCA) with R standard implementation was conducted in ESCC and paired normal tissues and batches. We were blinded to the group allocation during the experiments. The plots of the first two principal components are shown in Supplementary Fig. 22. Based on these figures, the samples are clearly divided into two groups, tumour and normal tissue, according to the second component. It is reassuring that none of the tumour or normal tissues had been contaminated, swapped or misplaced. None of the batches in tumour or normal tissues showed batch effects in PCA plots. We further performed regression analysis using batch ID and first 10 PCs and none of the regression coefficients was significant (all *P*>0.05), suggesting that technical batch effects in our data sets were reasonably small and thus unlikely to influence high-level analyses. Genes with *P*<0.05 (paired *t*-test) and expression fold change (>1.2 or <0.8) in tumours comparing with normal tissues were considered significantly differently expressed. To estimate the effect of copy-number change of each gene on its mRNA expression, Spearman's correlations were calculated between gene copy number and its corresponding mRNA expression. Correlations were considered significant and positive when *P*<0.05 and *r*>0.3.

### Comparison of ESCC with other types of cancer

To compare SNV pattern of ESCC with other types of cancers, WES data of 384 HNSCC and 179 LUSCC samples from the TCGA project and 141 EAC samples from the TumorPortal database^70^ were obtained. To compare CNV and RNA patterns, CNV and RNA data in 94 ESCC, 519 HNSCC, 512 LUSCC, 439 STAD and 79 EAC samples were obtained from the TCGA project. We used the Hierarchical Clustering to assess the SNV, CNV and RNA differences across different cancer types. PCA method was also used to assess the CNV and RNA differences. For the Hierarchical Clustering, mutation frequency or arm-level data were hierarchically clustered in R based on Euclidean distance using the Ward's method and RNA data were hierarchically clustered using the average linkage algorithm with 1 minus Pearson correlation coefficient as the dissimilarity measure.

### Cell lines

ESCC cell lines, KYSE30, KYSE150 and LUSCC cell line, NCI-H520, were cultured in RPMI 1640 medium supplemented with 10% foetal bovine serum (FBS). HNSCC cell line FaDu was cultured in Eagle's Minimum Essential Medium supplemented with 10% FBS. All cell lines were cultured in an atmosphere containing 5% CO_2_ at 37 °C. The KYSE series were generous gifts from Dr Y. Shimada at the Kyoto University. The LUSCC and HNSCC cell lines were purchased from the Institute of Basic Medical Sciences, Chinese Academy of Medical Sciences (Beijing, China). All cell lines used in this study were authenticated by short tandem repeat profiling and tested for absence of mycoplasma contamination (MycoAlert, Lonza Rockland).

### siRNA transfection

We mixed three siRNAs targeting the same gene as the siRNA-mix. Cells were transfected with siRNA-mix and Lipofectamine 2000 (Invitrogen) according to the manufacturer's instructions. In brief, 24 h before transfection, cells were plated onto a six-well plate (Corning, USA) at 30–50% confluence. The next day, cells with about 90% confluence were transfected with 2 μl Lipofectamine 2000 and 50 nM siRNA control or corresponding siRNA mix. After transfection, cells were incubated at 37 °C for 6 h and the medium was then replaced with fresh medium containing 10% FBS. After 48–72 h, cells were collected for further experiments. All siRNA oligos were purchased from GenePharma and their sequences are shown in Supplementary Data 16.

### Quantitative real-time PCR

Total RNA from cells was extracted with TRIzol (Invitrogen) according to the manufacturer's instruction. First-strand cDNA was synthesized using the PrimeScript 1st Strand cDNA Synthesis Kit (Takara). Relative RNA expression levels determined by quantitative real-time PCR were measured using the SYBR Green method on an ABI Prism 7,900 sequence detection system (Applied Biosystems) with SYBR Premix Ex Taq (Takara, Japan), 50 ng cDNA and 1 μM gene specific primers in a 25 μl reaction mixture. Reactions were performed with an initial 30-s denaturation step at 95 °C, followed by 40 cycles of 95 °C for 5 s and 60 °C for 30 s. Independent experiments were done in triplicate. *GAPDH* was employed as an internal control. All gene specific primers are shown in Supplementary Table 7.

### Cell proliferation and migration or invasion assays

Cells were plated in 96-well plates and each well contains 2,000 cells in 200 μl cell suspension after transfection with siRNA-mix. Cell numbers were measured with CCK-8 (Dojindo) at 24, 48, 72 and 96 h after incubation. The migration and invasion ability of each cell line was evaluated, respectively, using the 24-well chambers (8 μm pore size; Corning). For migration assays, 5–10 × 10^4^ cells in 150 μl serum-free RPMI 1640 medium were added into the upper chamber. For invasion assays, cells were added after coating filters with matrigel. RPMI 1640 medium with 20% FBS was used as a chemoattractant in the lower chamber. After 24-h incubation at 37 °C, cells were fixed in methanol and stained with 0.5% crystal violet. After removing cells on the upper chamber, cells that migrated to the bottom side of the membrane were counted. Comparison with siRNA control was performed with unpaired *t*-test.

### Data availability

The raw sequencing data of this study have been deposited in the Genome Sequence Archive of Beijing Institute of Genomics, Chinese Academy of Sciences (http://gsa.big.as.cn/) with accession number PRJCA000354. Data that support the findings of this study are available from TCGA database (http://cancergenome.nih.gov) and TumorPortal (http://www.tumorportal.org)

## Additional information

**How to cite this article:** Chang, J. *et al*. Genomic analysis of oesophageal squamous-cell carcinoma identifies alcohol drinking-related mutation signature and genomic alterations. *Nat. Commun.* **8**, 15290 doi: 10.1038/ncomms15290 (2017).

**Publisher's note**: Springer Nature remains neutral with regard to jurisdictional claims in published maps and institutional affiliations.

## Supplementary Material

Supplementary Information Supplementary Figures and Supplementary Tables

Supplementary Data 1 Select characteristics of individuals with ESCC in this study

Supplementary Data 2 Summary of statistics for whole-genome sequencing

Supplementary Data 3 Somatic mutations in exonic regions identified by whole-genome sequencing

Supplementary Data 4 Significantly mutated genes in ESCC and other cancers

Supplementary Data 5 Associations between mutation status of 20 ESCC driver genes and clinical characteristics

Supplementary Data 6 Genes in the focal amplification regions

Supplementary Data 7 Genes in the focal deletion regions

Supplementary Data 8 Fifty-seven focal CNV regions identified in ESCC

Supplementary Data 9 All genes with CNVs

Supplementary Data 10 Results of gene-drug interaction analyzed using DGIdb

Supplementary Data 11 586 genes with CNVs in three squamous cell cancers

Supplementary Data 12 Comparison of the expression of genes with and without SV

Supplementary Data 13 Genes involved in pathways

Supplementary Data 14 Pathway analysis of driver genes in ESCC

Supplementary Data 15 Sanger sequencing validation of somatic mutations detected by whole-genome sequencing in ESCC

Supplementary Data 16 siRNA sequences used in this study
