# Supplementary material for: Genomic analysis of oesophageal squamous-cell carcinoma identifies alcohol drinking-related mutation signature and genomic alterations
Source: Nat Commun. 2017 May 26;8:15290. doi: 10.1038/ncomms15290 (PMC5477513; doi:10.1038/ncomms15290)
Supplement: Supplementary Information — Supplementary Figures and Supplementary Tables [file ncomms15290-s1.pdf]

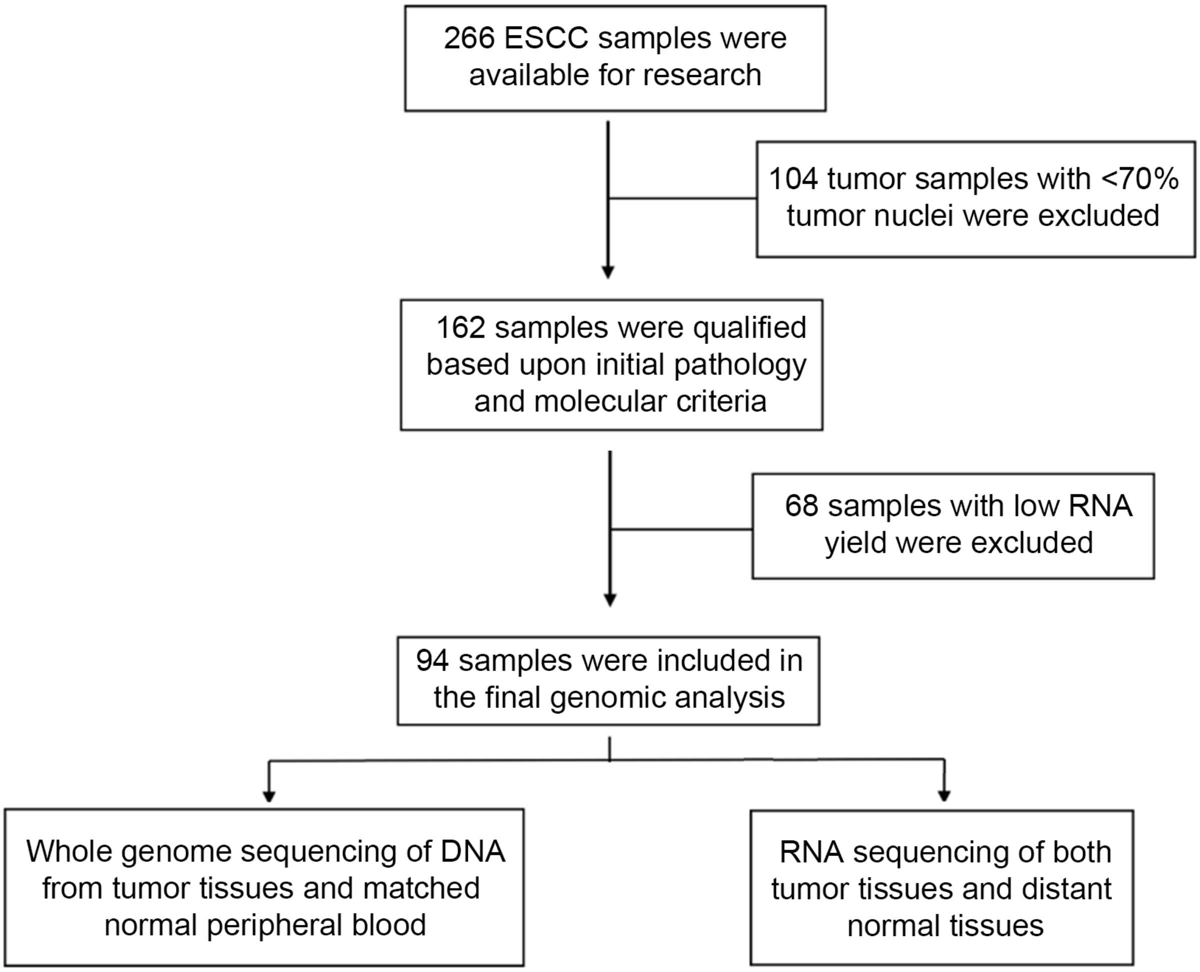

Supplementary Figure 1. Flow chart of eligibility of ESCC samples.

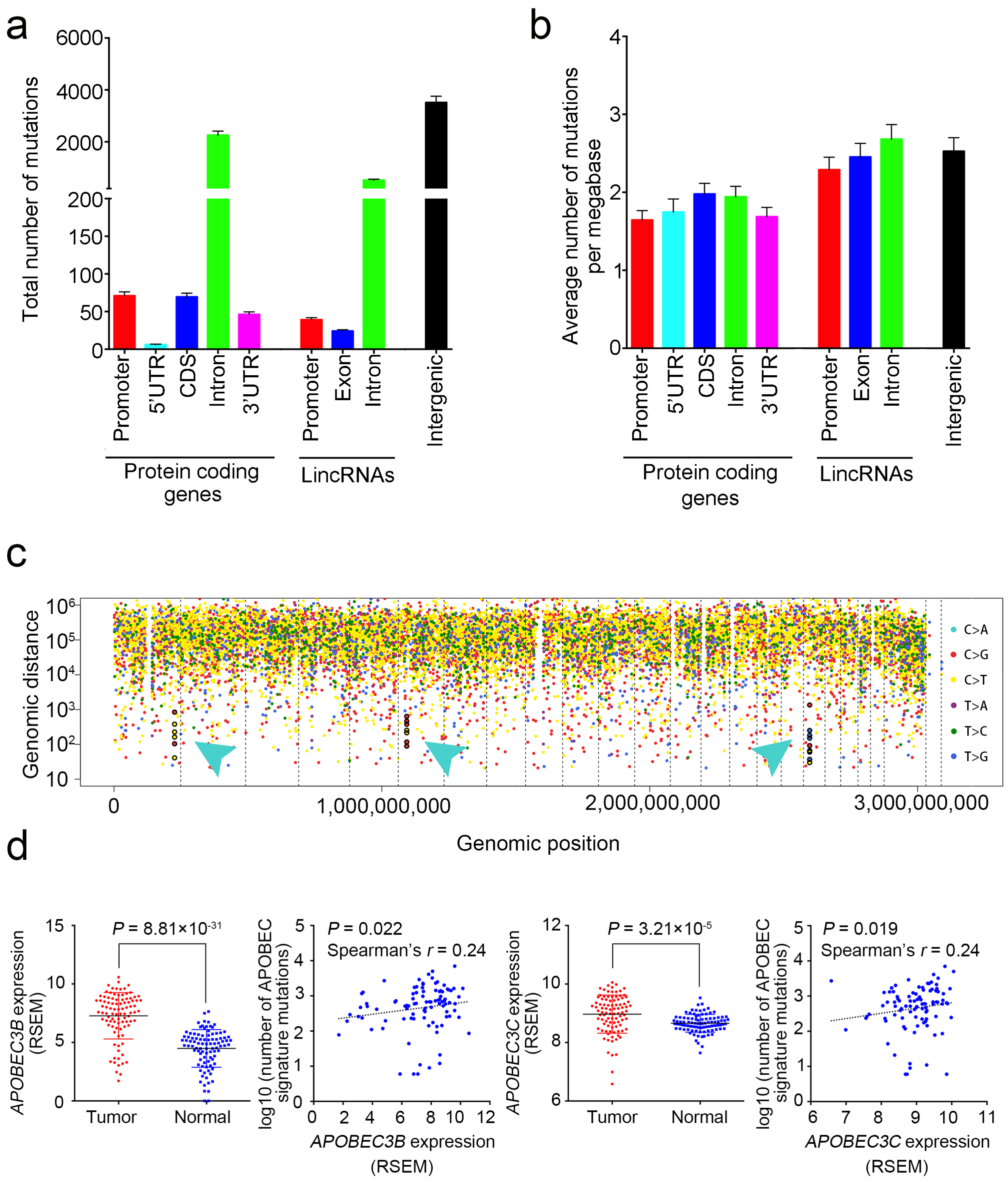

Supplementary Figure 2. Mutation spectrum of 94 ESCC detected by whole-genome sequencing. (**a**) The number of mutations in different genomic regions of 94 ESCC samples. Data represent mean ± s.e.m. of mutations per sample (y axis) by type of genomic regions (x axis). CDS, coding sequence. (**b**) The number of mutations per megabase in different genomic regions of 94 ESCC samples. Data represent mean ± s.e.m. of mutations (y axis) by type of genomic regions (x axis). (**c**) One example of kataegis of ESCC_191. The 'rainfall' plot represents an individual ESCC sample in which each dot represents a single somatic mutation ordered on the horizontal axis according to its position in the human genome. The vertical axis denotes the genomic distance of each mutation from the previous mutation. (**d**) Comparison of *APOBEC3B* and *APOBEC3C* mRNA expression in tumor and normal samples (*P*-values were obtained by Student's *t*-test) and correlation of *APOBEC3B* and *APOBEC3C* mRNA expression and number of APOBEC signature mutations. The *APOBEC3B* or *APOBEC3C* mRNA expression (RSEM) was added by 1 and then log2 transformed.

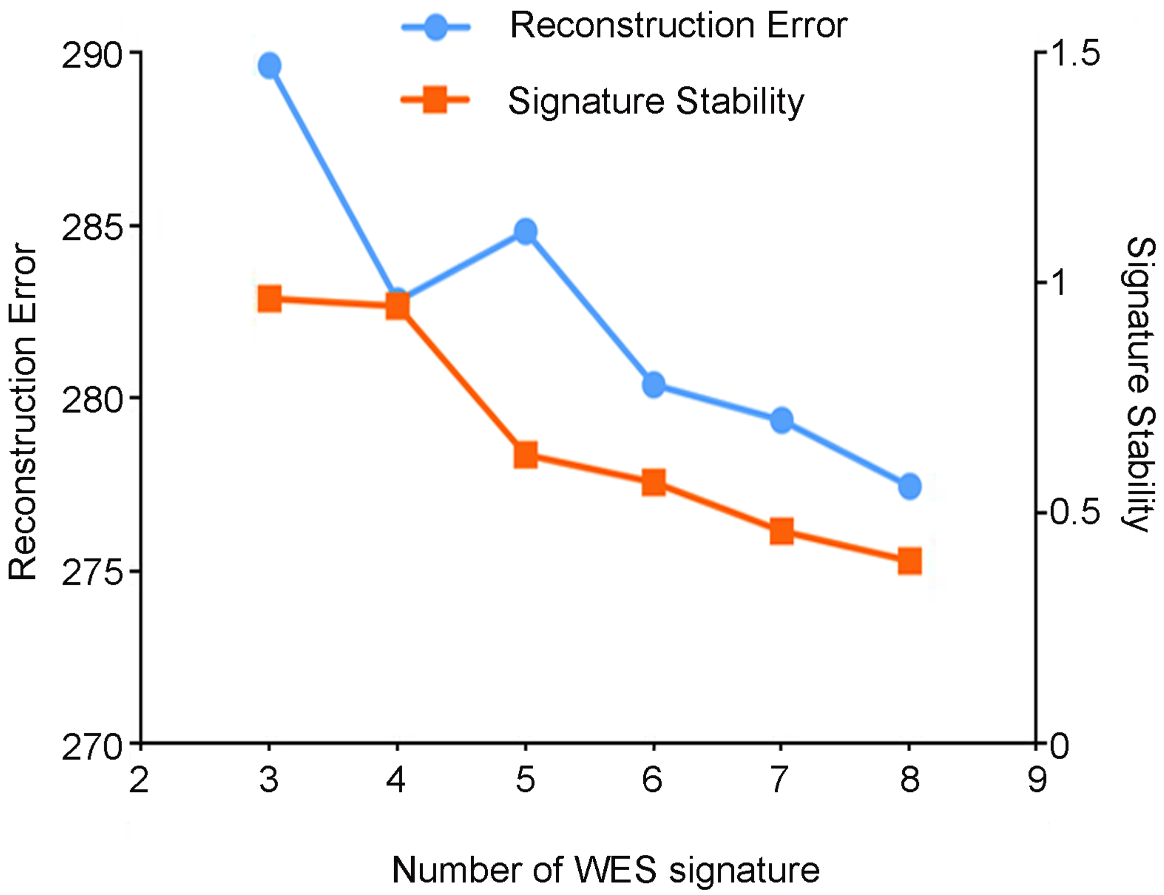

Supplementary Figure 3. Signature stability and reconstruction error of non-negative-matrix factorization analysis. Non-negative-matrix factorizations with different numbers of signatures were tried, from three to eight. The y axis represents reconstruction error (*left*) and signature stability (*right*).

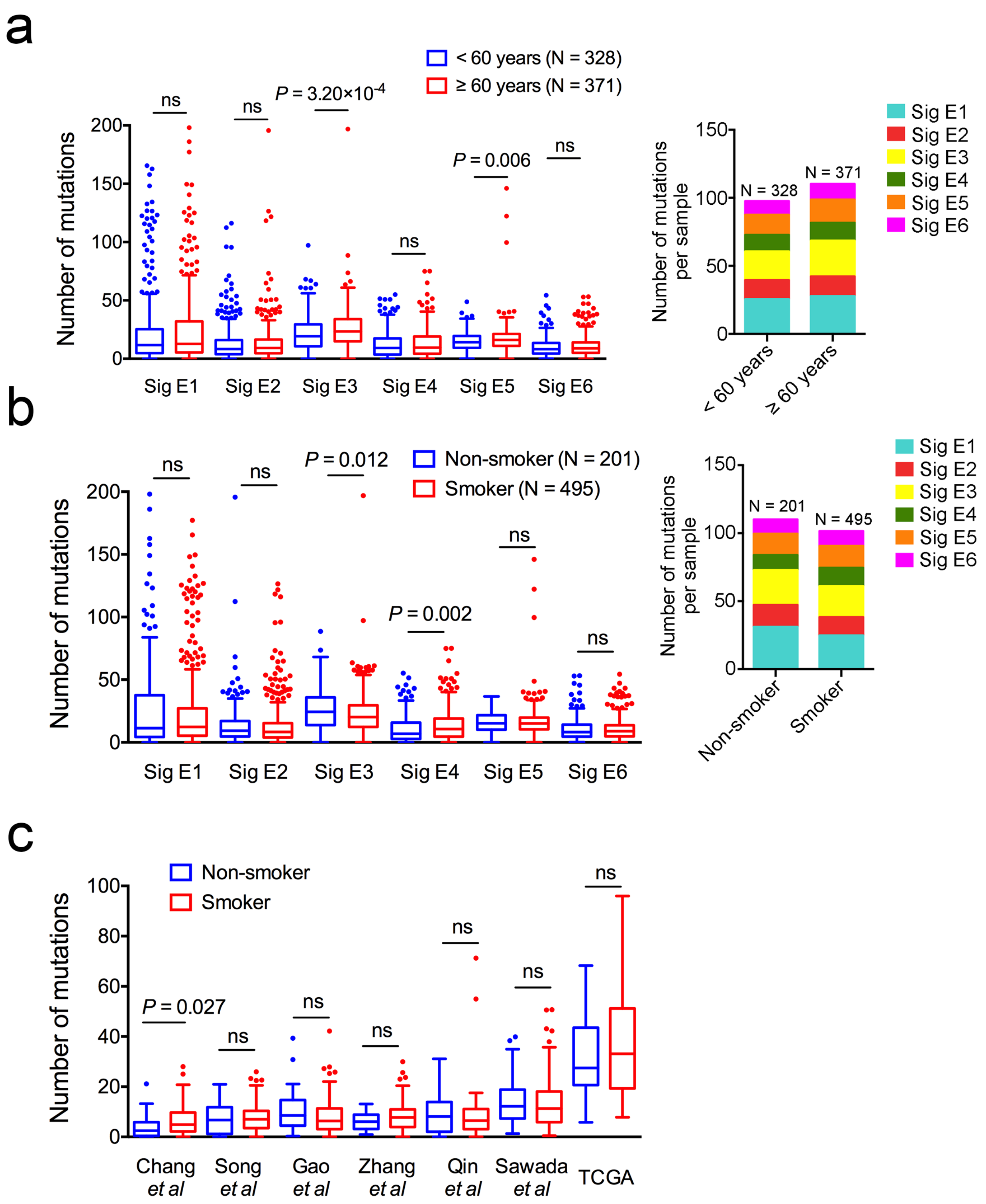

Supplementary Figure 4. Associations between mutational signatures and age at ESCC diagnosis or smoking status. The y axis denotes number of mutations. Mutational Signatures E3 and E5 were significantly associated with age at ESCC diagnosis (**a**), while Mutational Signatures E3 and E4 were significantly associated with tobacco smoking (**b**). No significant association of mutation number was detected in other previously reported studies (**c**). Data are presented in Tukey's boxplot. The line in the middle of the box is plotted at the median while the upper and lower hinges represent 25th and 75th percentiles. Whiskers indicate1.5 times interquartile range (IQR) and values greater than it are plotted as individual points. The minima and maxima are the lowest datum still within 1.5 IQR of the lower quartile and the highest datum still within 1.5 IQR of the upper quartile. *P*-values were obtained by unpaired Wilcox rank-sum test. ns, not significant.

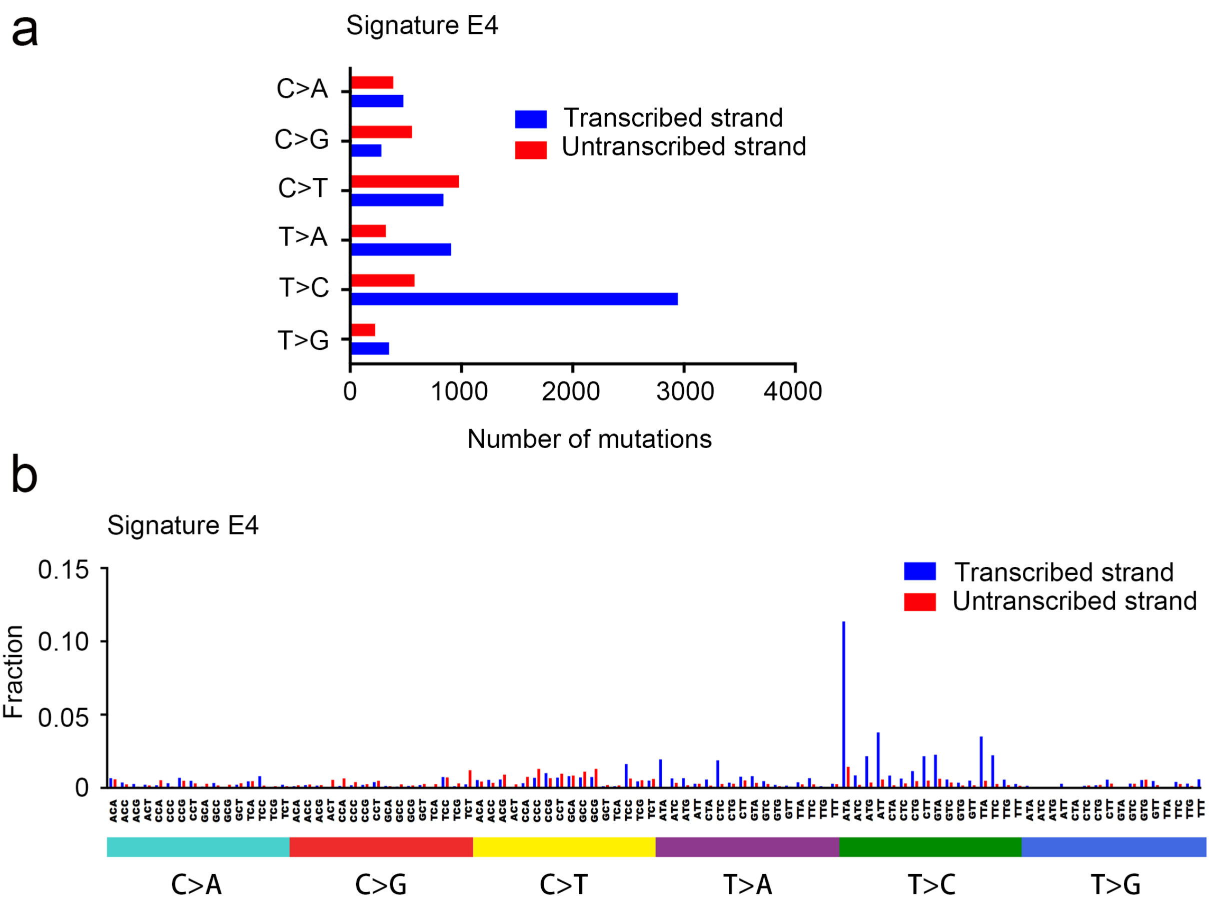

Supplementary Figure 5. Transcriptional bias of mutational Signature E4. (**a**) Mutations are clustered into six types (y axis) and number of mutations in each type (x axis) is shown. Mutations on the transcribed strand are displayed in blue while mutations on the untranscribed strand are displayed in red. (**b**) Mutational signature is displayed using a 192-substitution classification incorporating the substitution type, the sequence context immediately 5' and 3' to the mutated base and whether the mutated base is on the transcribed or untranscribed strand (x axis). The panels for each of the six types of substitutions as well as the mutated base are displayed in different colors. The mutation fractions for each type are shown in y axis. Mutations on the transcribed strand are displayed in blue while mutations on the untranscribed strand are displayed in red.

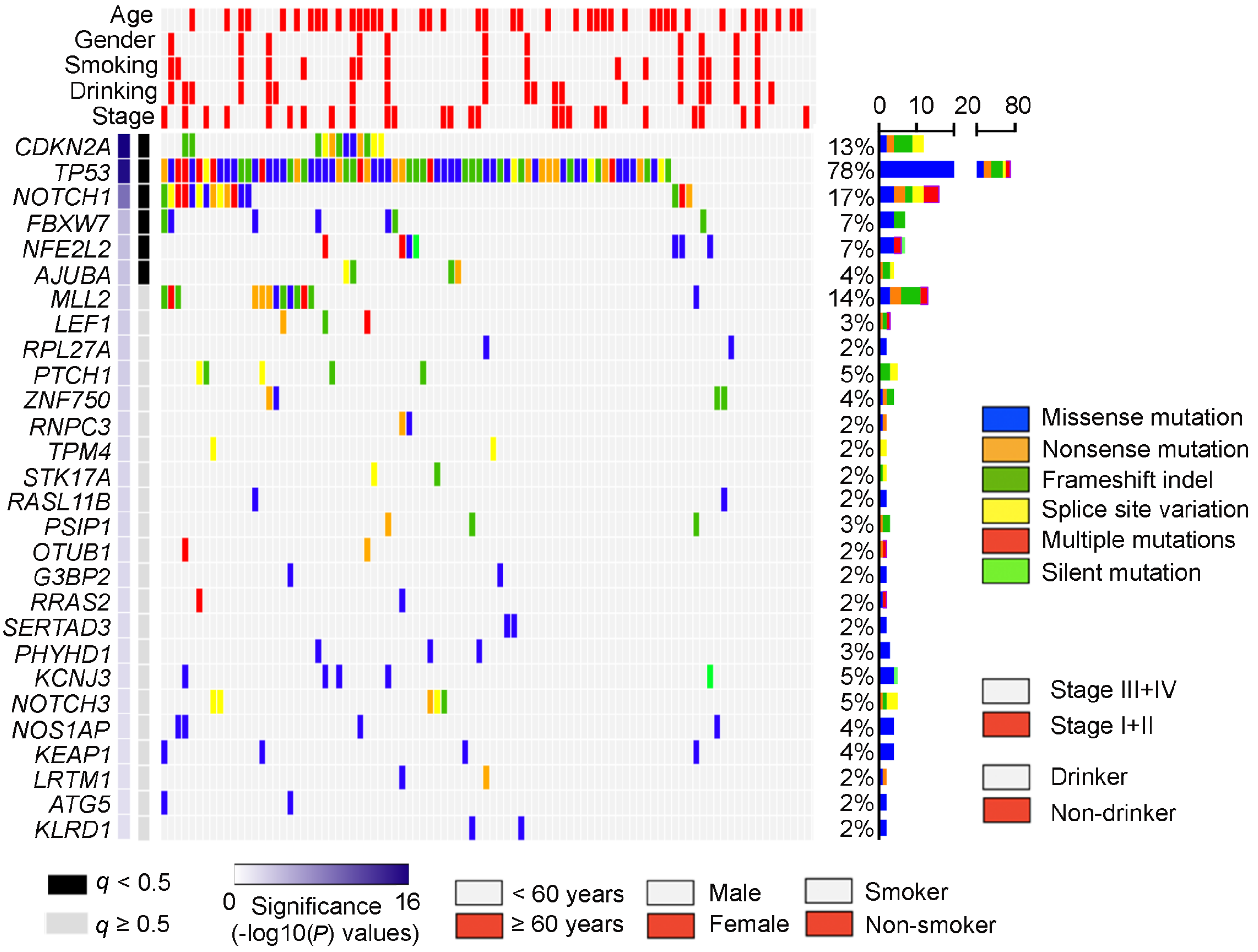

Supplementary Figure 6. Mutation frequencies and significantly mutated genes in 94 ESCC detected by whole-genome sequencing. Significantly mutated genes (identified using the MutSigCV algorithm; FDR *q*<0.5) ordered by *q* value; additional genes (*P*<0.01) with trends towards significance are also shown. Samples are arranged to emphasize mutual exclusivity among mutations. Each column denotes an individual tumor, and each row represents a gene. *Top*, key clinical parameters of each examined case. *Right*, mutation percentage (x axis) and number of mutations (y axis) for each gene in 94 ESCC. Clinical characteristics and mutation types are shown by color as indicated.

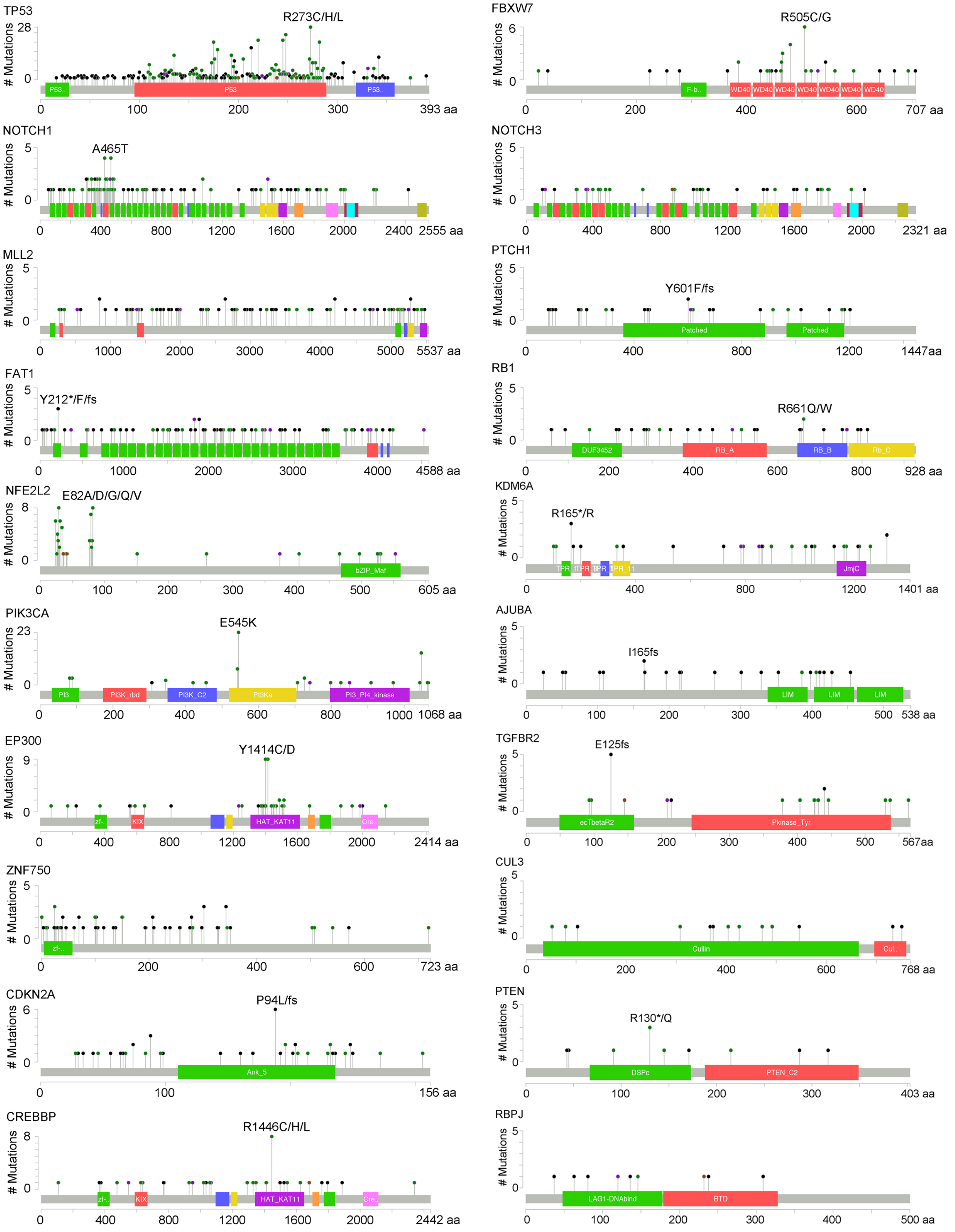

Supplementary Figure 7. Schematics of protein alterations for 20 significantly mutated ESCC driver genes. Known functional domains of each protein are mapped from the UniProt. The predicted impact of mutations is shown by a filled colored circle and stick. The color code is as follows: green = missense, red = frame shift indel & nonsense & splice site, black = in frame indel, grey = silent, and purple = multiple mutations. The height of the lollipop is the number of mutations at a position. The most frequent mutation is labeled with its amino acid change. The figures are created using the cBioPortal mutation mapper with manual coloring of the mutations.

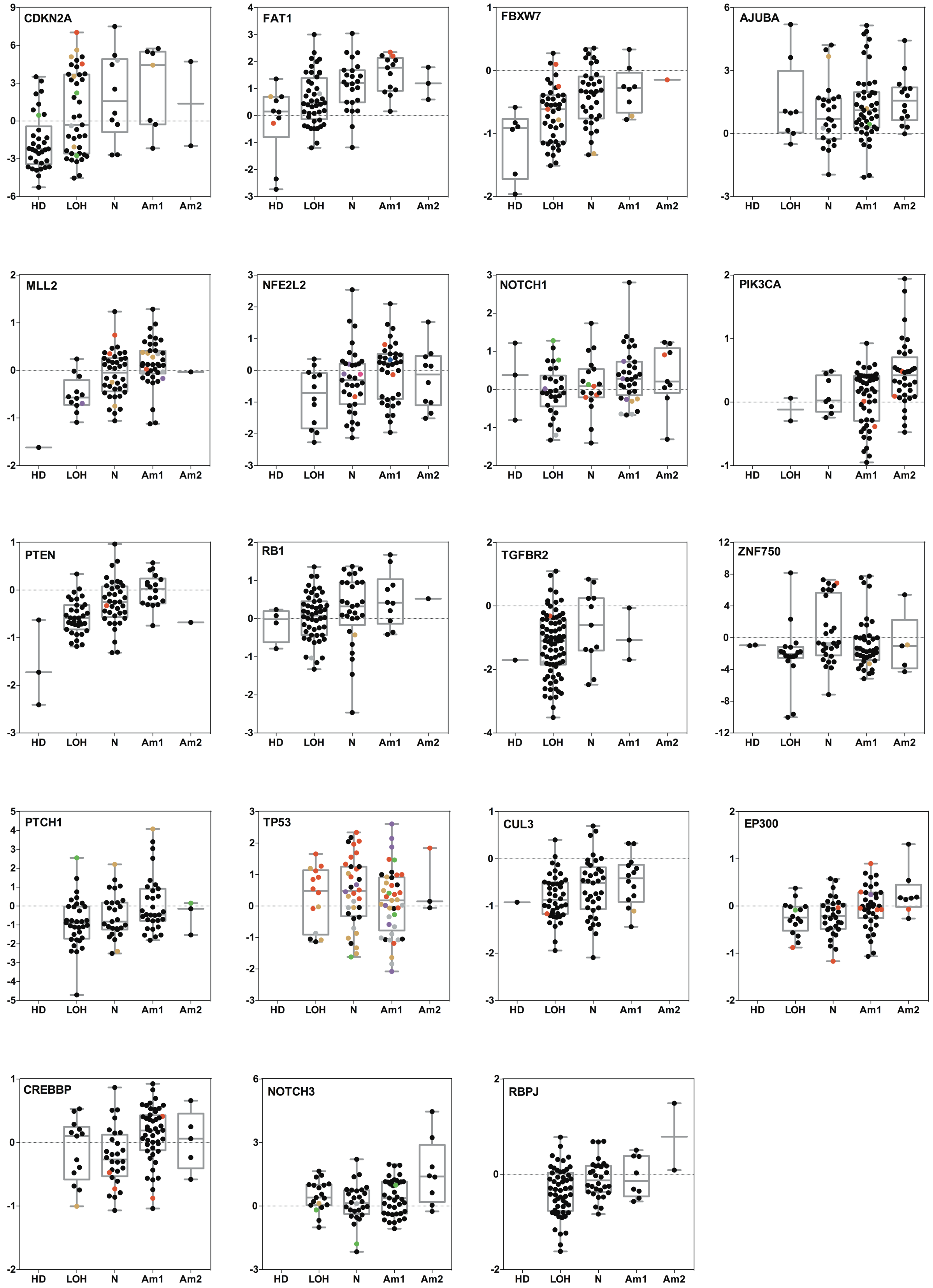

Supplementary Figure 8. Integrative analysis of mutation type, copy number and mRNA expression of predicted driver genes in ESCC. Nineteen driver genes (except *KDM6A*, located on chromosome X) are ordered by *q*-value. x axis represents discrete DNA copy number values while y axis represents mRNA expression measurements. Mutations are indicated as follows: red = missense, grey = nonsense, yellow ochre = frame shift indel, blue = in frame indel, green = splice site, hot pink = silent and purple = multiple mutations in single sample. Black indicates wild type sequence. DNA copy number status is coded as follows: HD = homozygous deletion, LOH = heterozygous deletion, N = copy neutral, Am1 = single copy gain, Am2 = multiple copy gain.

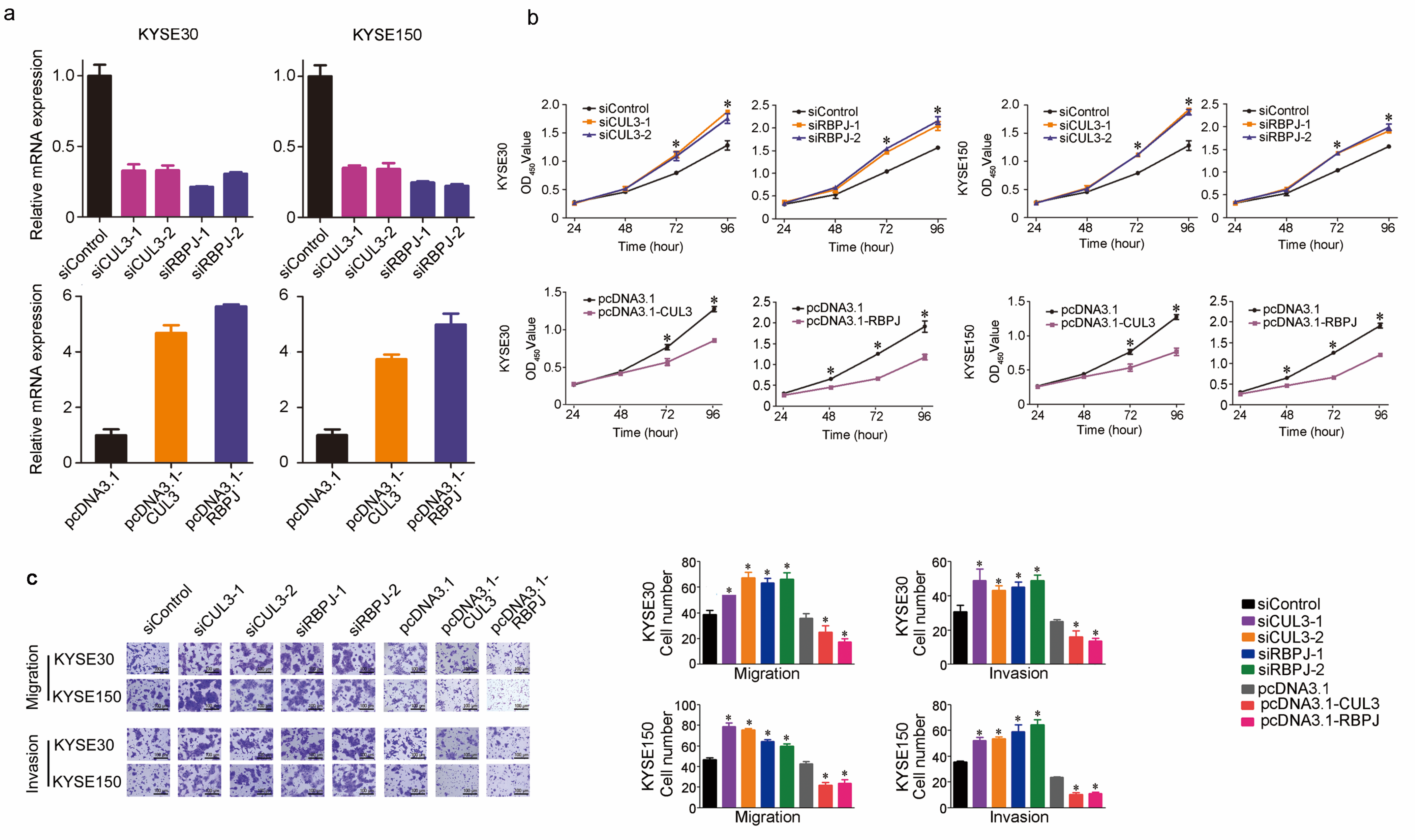

Supplementary Figure 9. Functional analysis of significantly mutated *CUL3* and *RBPJ* genes. (**a**) Knockdown or overexpression of the *CUL3* and *RBPJ* genes in ESCC cells. Significant knockdown of the expression of the two genes by siRNA (*upper panel*) and ectopic overexpression of the two genes by transfection of pcDNA3.1-CUL3 and pcDNA3.1-RBPJ in ESCC cells (*lower panel*). The data represent mean ± s.e.m of mRNA expression from three independent experiments and each had three replications. (**b**) Effects of knockdown or overexpression of *CUL3* and *RBPJ* on ESCC cell proliferation. Data represent mean ± s.e.m of OD_{450} values from three independent experiments and each had three replications. *, *P*<0.01 compared with control by Student's *t*-test. (**c**) Effects of knockdown or overexpression of *CUL3* and *RBPJ* on ESCC cell migration and invasion (*upper panel*). Data (*lower panel*) represent mean ± s.e.m. of cell number from three independent experiments and each had duplication. *, *P*<0.01 by Student's *t*-test as compared with siRNA control or pcDNA3.1 vector control. Scale bars,100 μm.

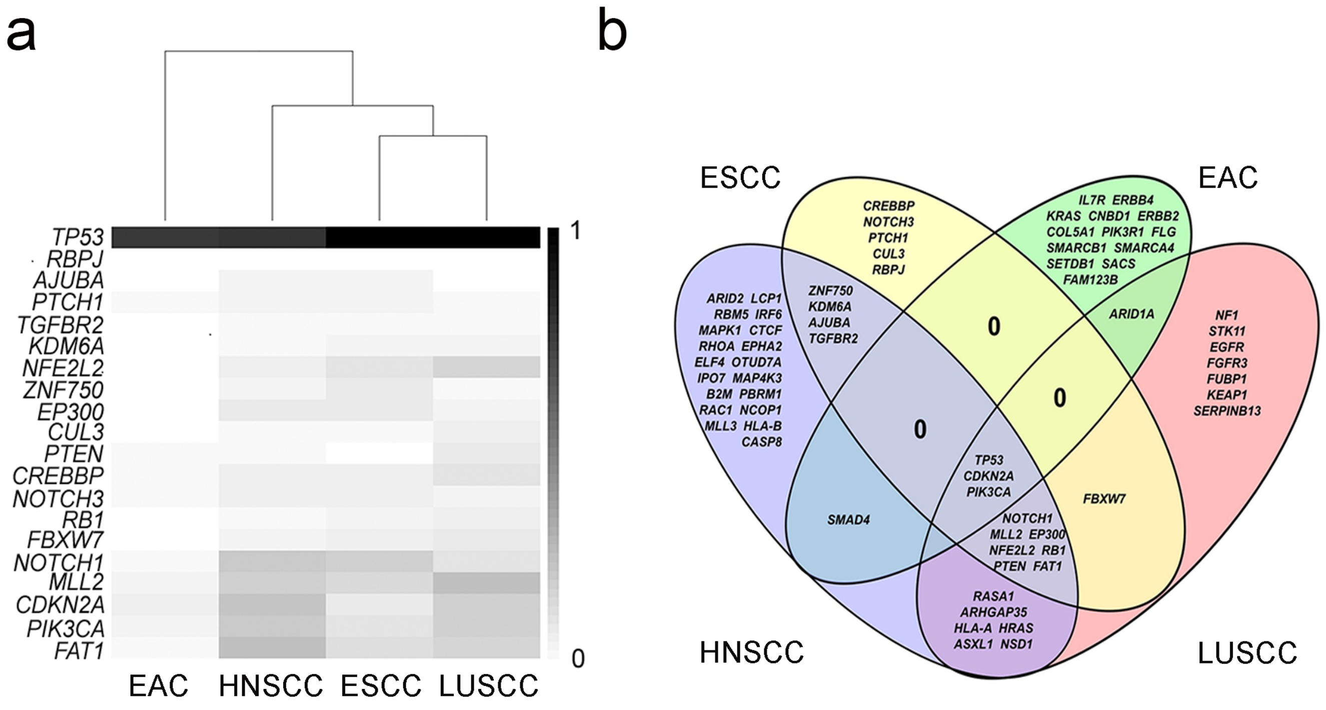

Supplementary Figure 10. Comparison of mutations in ESCC and other types of cancer. (**a**) Hierarchical clustering using the mutation frequency of 20 ESCC driver genes in ESCC, HNSCC, LUSCC and EAC. (**b**) Significant driver gene that is unique or common in different cancer types. Driver genes in HNSCC, LUSCC and EAC are derived from tumor portal (http://www.tumorportal.org).

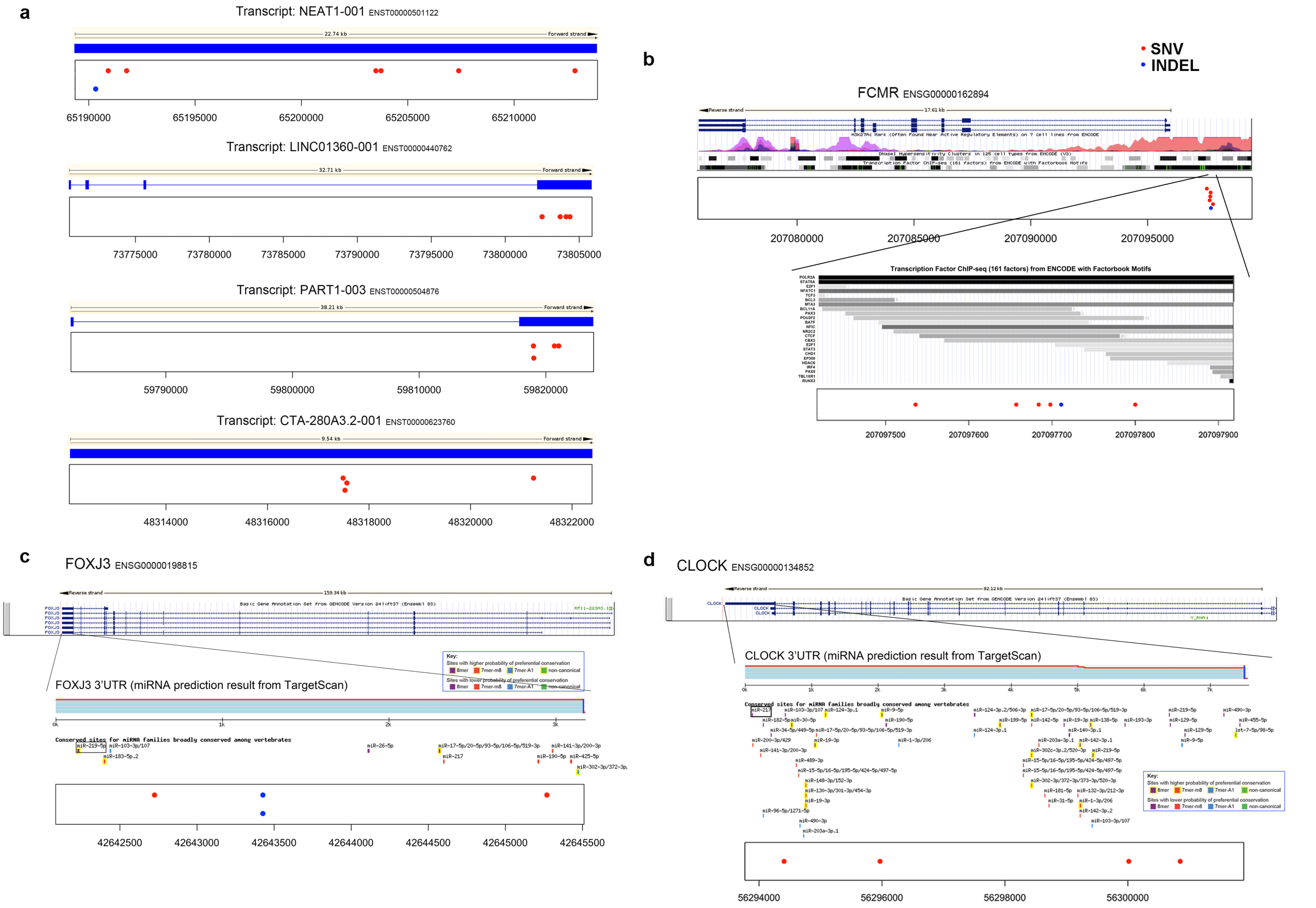

Supplementary Figure 11. Significant mutations in the non-coding regions in ESCC. (**a**) mutations in four long non-coding RNAs. (**b**) Mutations in the promoter region of the *FCMR* gene. (**c & d**) Mutations in the 3'-untraslated regions (3'UTR) of the *FOXJ3* and *CLOCK* genes.

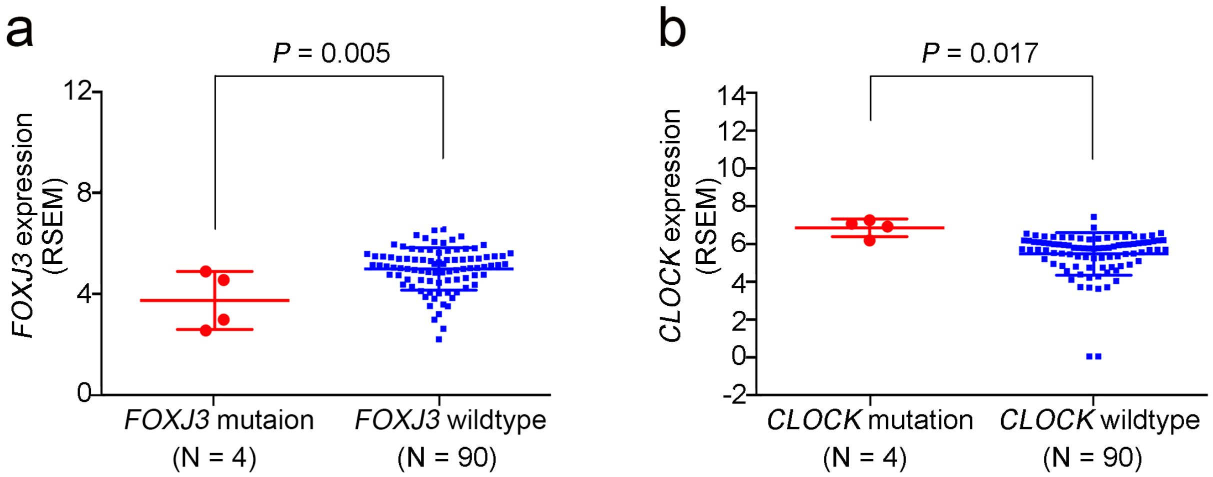

Supplementary Figure 12. Effects of mutations in the 3'-untranslated regions of *FOXJ3* (a) and *CLOCK* (b) on their mRNA expression. Data are mean ± s.d. and the *P*-values were obtained by unpaired Student's *t*-test.

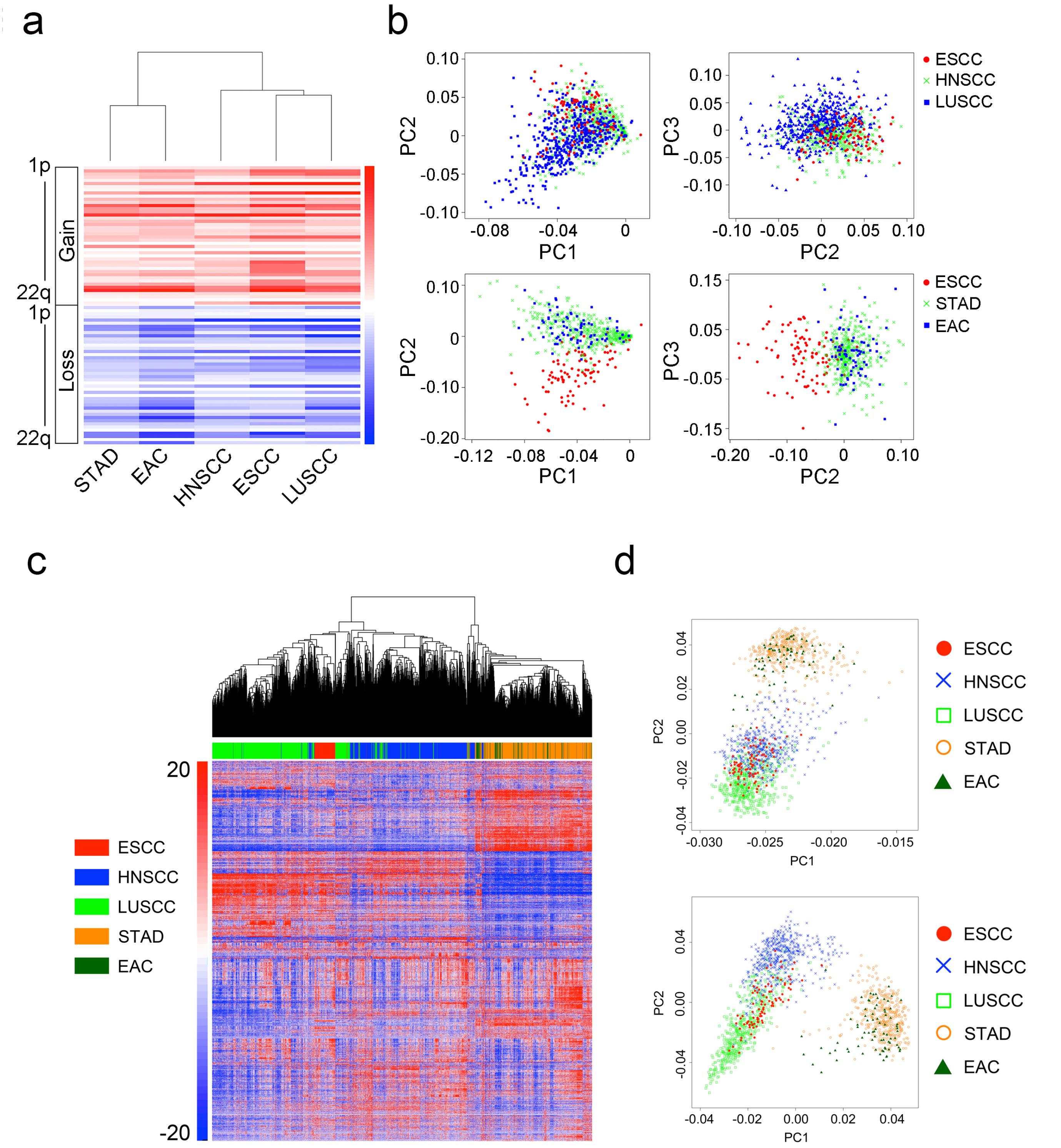

Supplementary Figure 13. Hierarchical clustering and PCA analysis of copy number alteration and mRNA expression in ESCC and other cancers. Data are from 94 ESCC, 519 HNSCC, 512 LUSCC, 439 STAD and 79 EAC samples. (**a**) Hierarchical clustering were performed using the arm-level alteration frequency with Euclidean distance under the Ward's method for different cancer types (x axis). The heat map shows the frequency of chromosomal copy gains (upper) and losses (lower) ordered from chromosome 1p to 22q (y axis). (**b**) PCA was performed using whole genome copy number profiles. (**c**) The expression of top 6,000 most variable genes was obtained and log2 transformed followed by gene mean centering. Hierarchical clustering was performed using the average linkage algorithm with 1 minus Pearson correlation coefficient as the dissimilarity measure. (**d**) PCA was performed using the log2 transformed expression of the top 6,000 most variable genes.

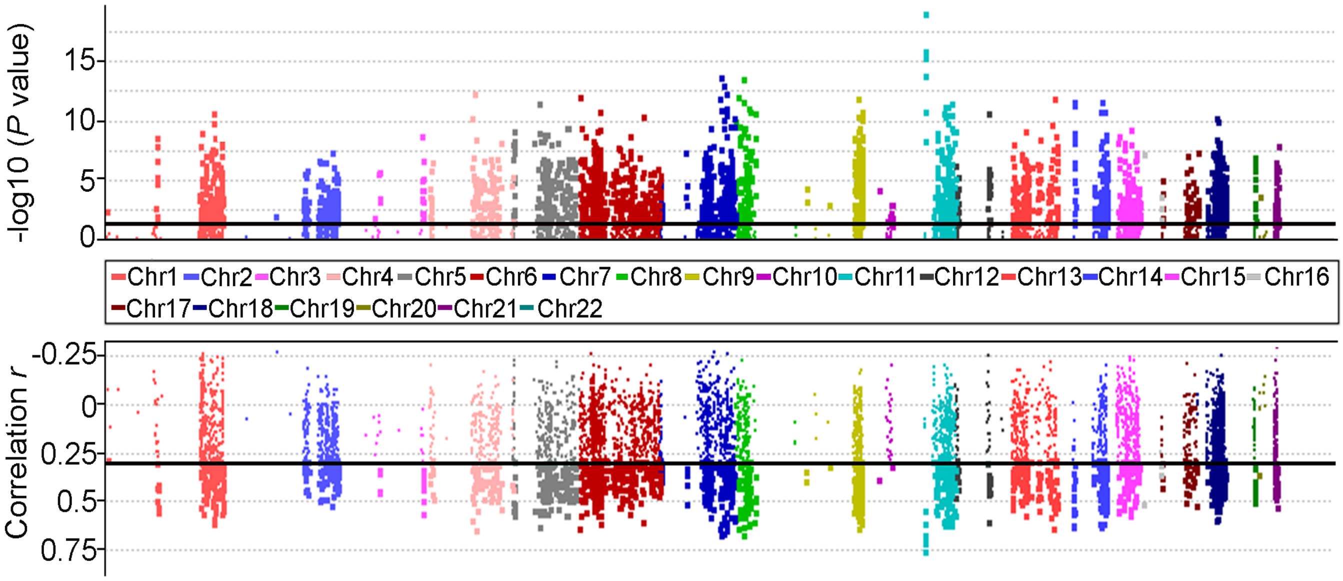

Supplementary Figure 14. Correlation between copy number change and mRNA expression in 57 significant peak regions of focal genomic alterations. Spearman's correlation *P* (*upper*) and *r* values (*lower*) between copy number and expression of genes (y axis) are shown. Genes are ordered by their chromosome positions (x axis). Black lines indicate *P*=0.05 (*upper*) and *r*=0.3 (*lower*).

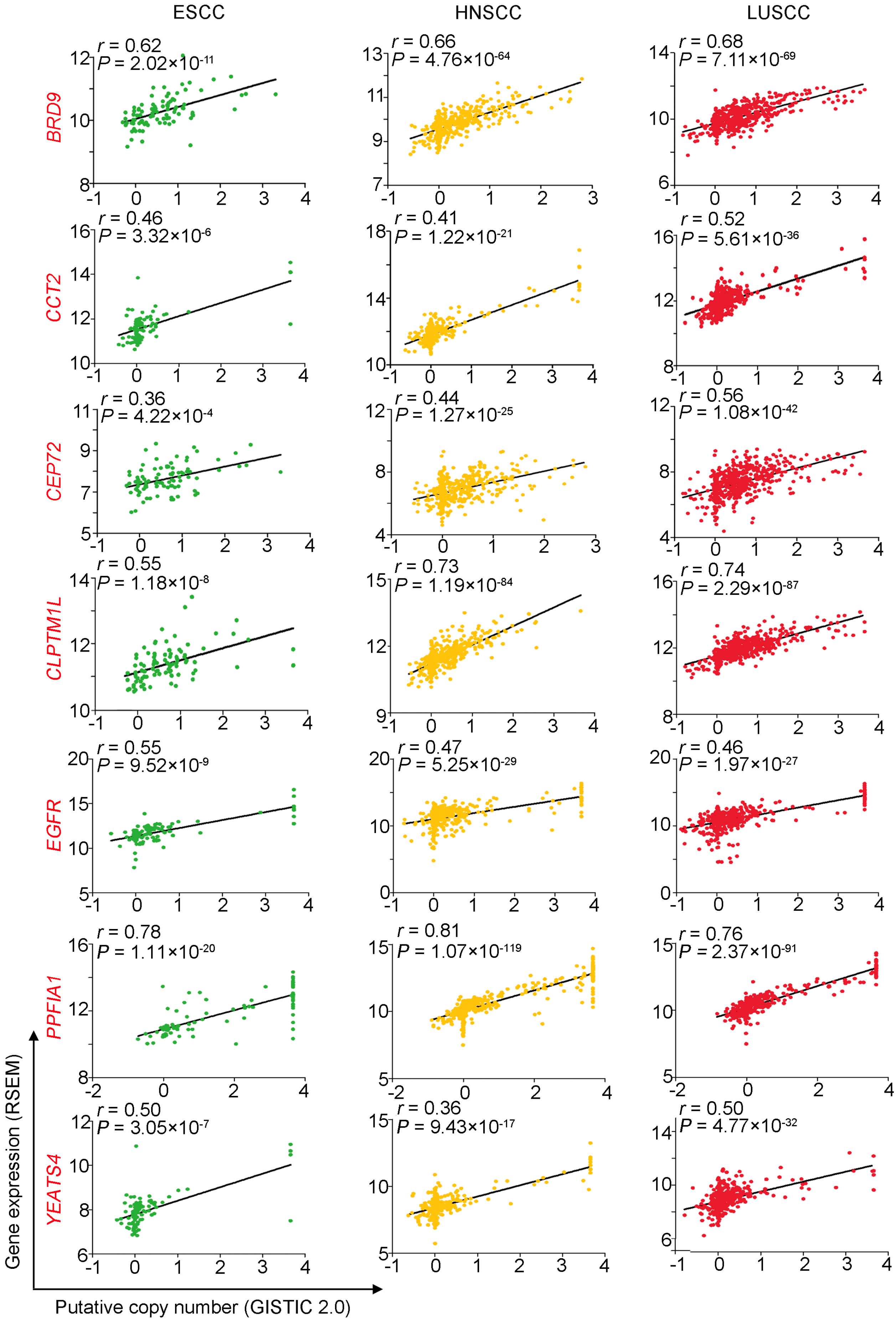

Supplementary Figure 15. Correlations between copy number change and mRNA expression of 7 genes commonly amplified in three squamous cell cancers. Gene expression (RSEM) are shown in y axis. Putative copy number in x axis (see Supplementary Data 11) are estimated by the GISTIC 2.0 (all_data_by_genes.txt) with copy number as a continuous variable.

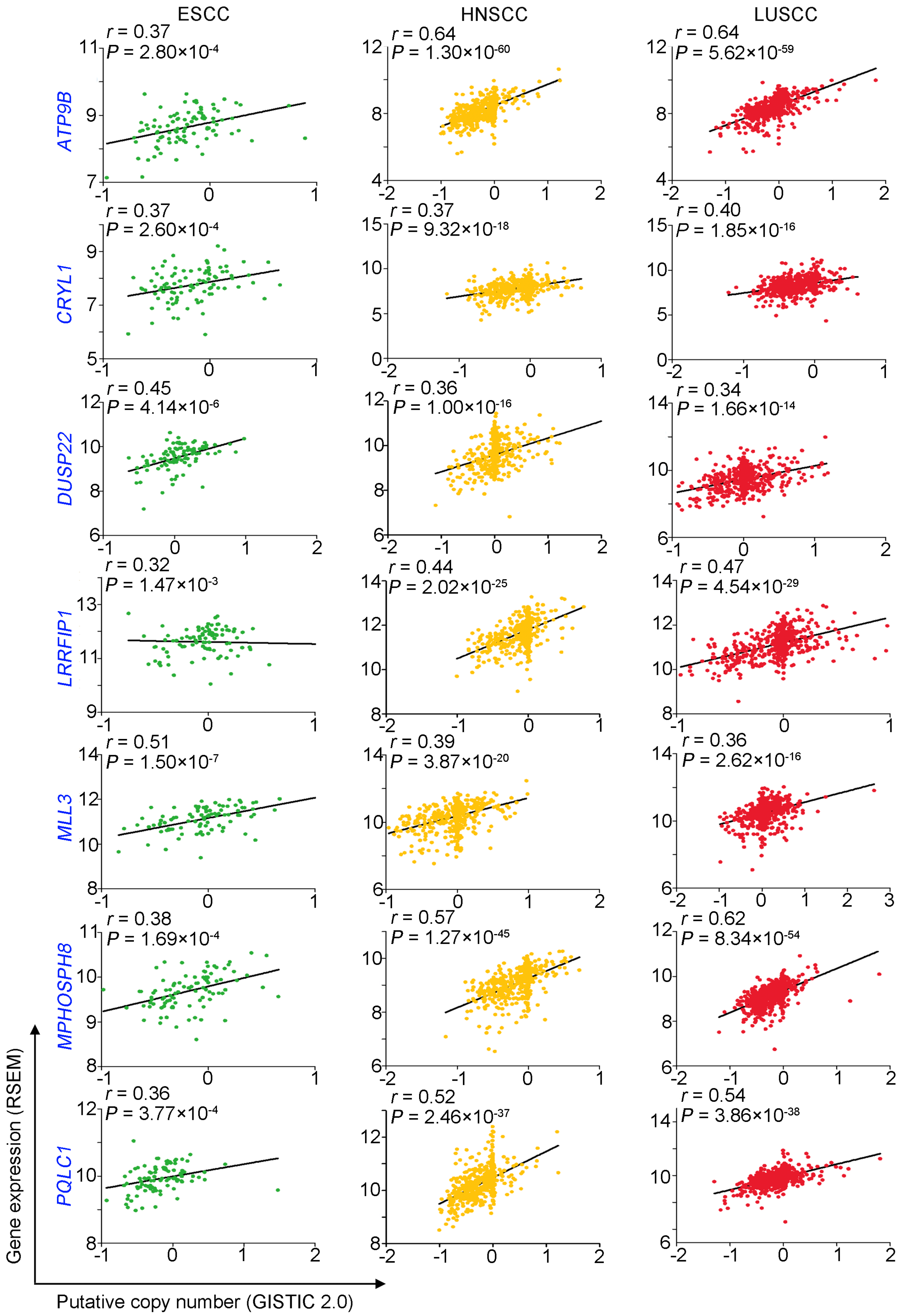

Supplementary Figure 16. Correlations between copy number change and mRNA expression of 7 genes commonly deleted in three squamous cell cancers. Gene expression are showed in y axis. Putative copy number in x axis (see Supplementary Data 11) are estimated by the GISTIC 2.0 (all_data_by_genes.txt) with copy number as a continuous variable.

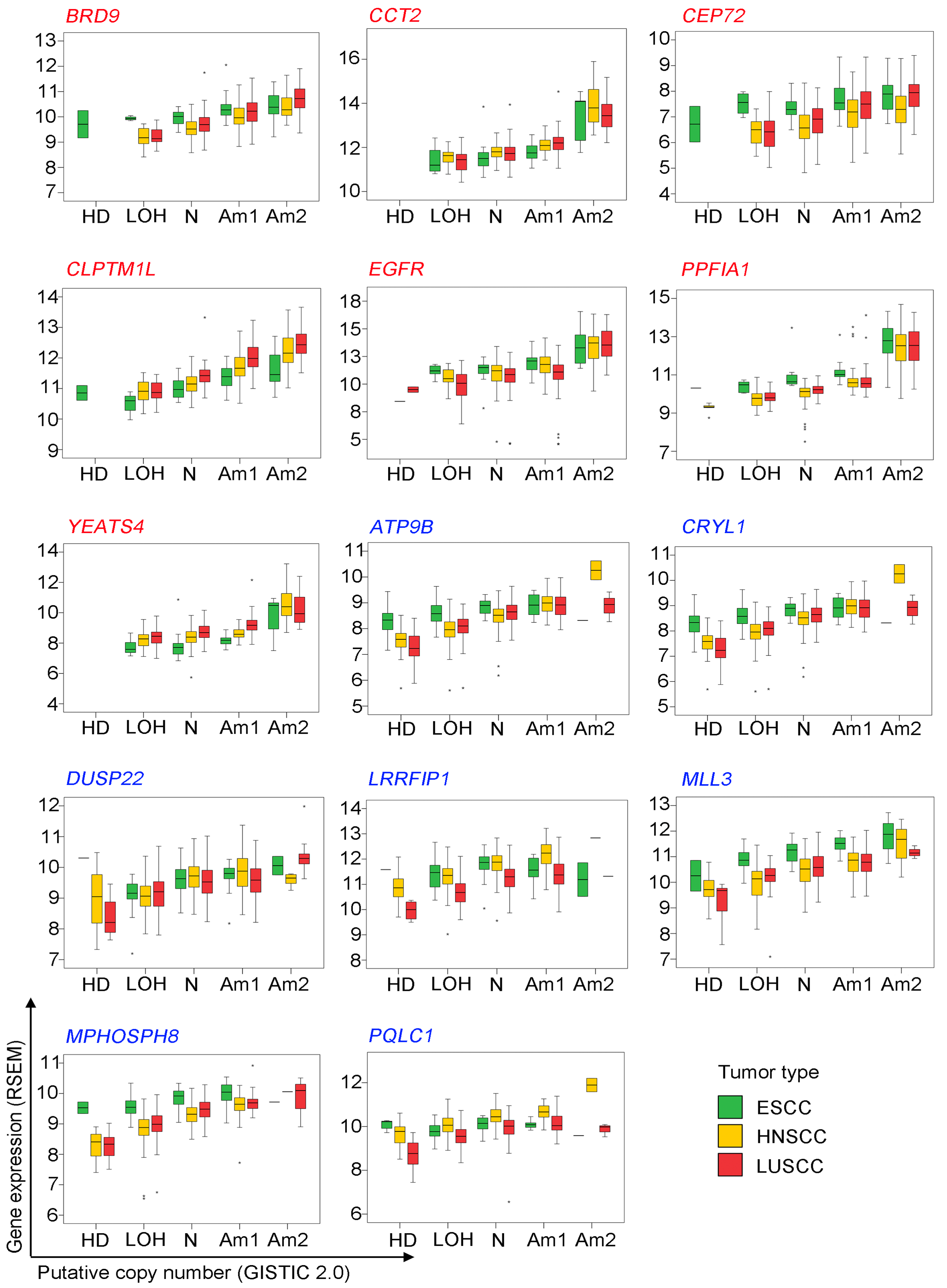

Supplementary Figure 17. Correlations between copy number change and mRNA expression of 14 genes commonly observed in three squamous cell cancers. Gene expression are shown in y axis. Putative copy number in x axis (see Supplementary Data 11) are estimated by the GISTIC 2.0 (all_thresholded.by_genes.txt) with copy number as a categorical variable. Tumor types are denoted by color as indicated. Data are presented in Tukey's boxplot. The line in the middle of the box is plotted at median while the upper and lower hinges represent 25th and 75th percentiles. Whiskers indicate 1.5 times interquartile range (IQR) and values greater than it are plotted as individual points. The minima and maxima are the lowest datum still within 1.5 IQR of the lower quartile and the highest datum still within 1.5 IQR of the upper quartile.

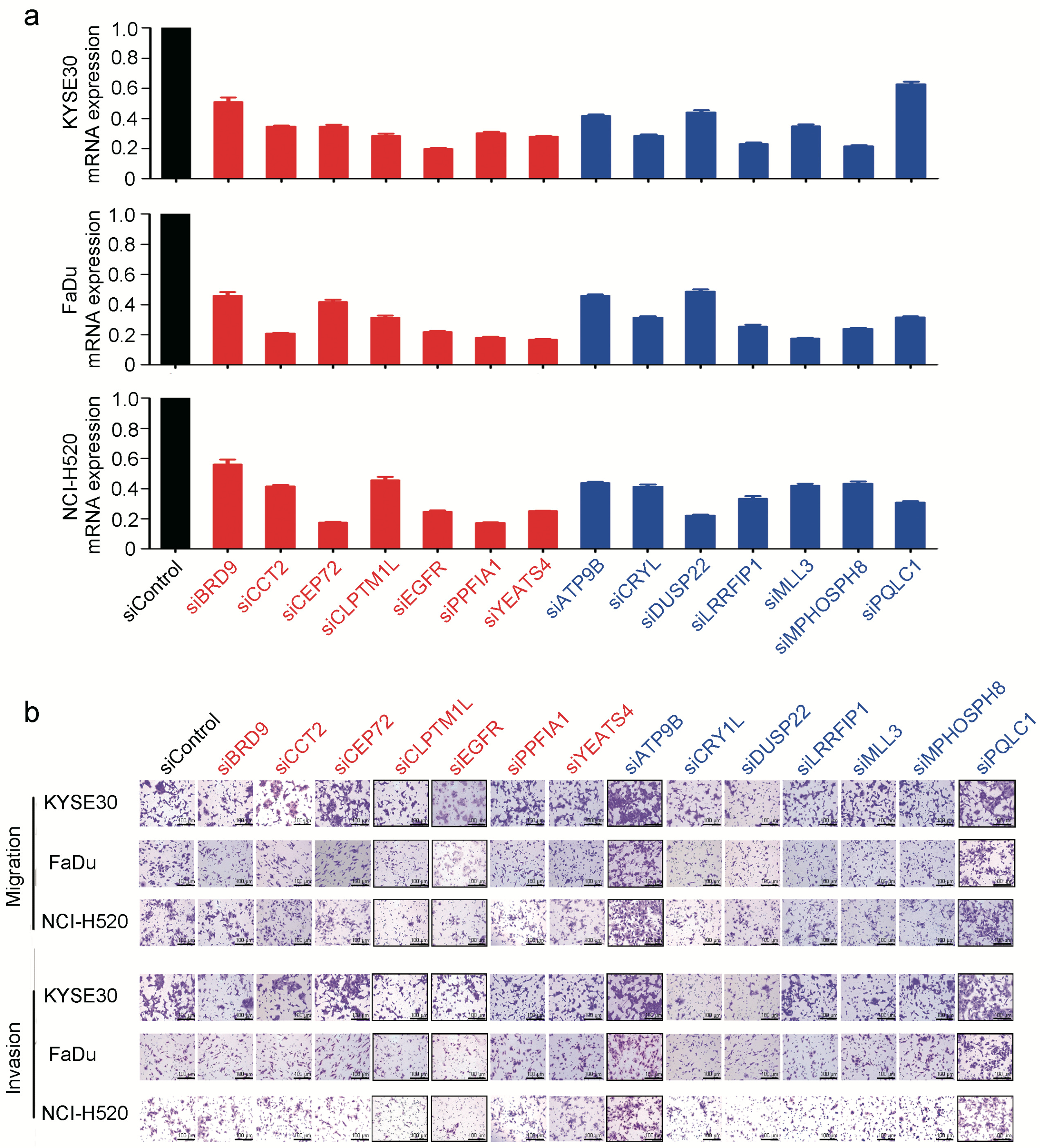

Supplementary Figure 18. Functional analysis of 14 genes affected by copy number alteration commonly observed in three types of squamous cell cancer. (**a**) Knockdown of the expression of the 14 genes in three SCC cell lines by siRNA. Data represent mean ± s.e.m. of mRNA expression from three independent experiments and each had triplication. (**b**) Effects of knockdown of the 14 genes on SCC cell migration and invasion. Shown are typical cell migration and invasion pictures; quantitative data are presented in Figure 3c in the main text. Scale bars,100 μm.

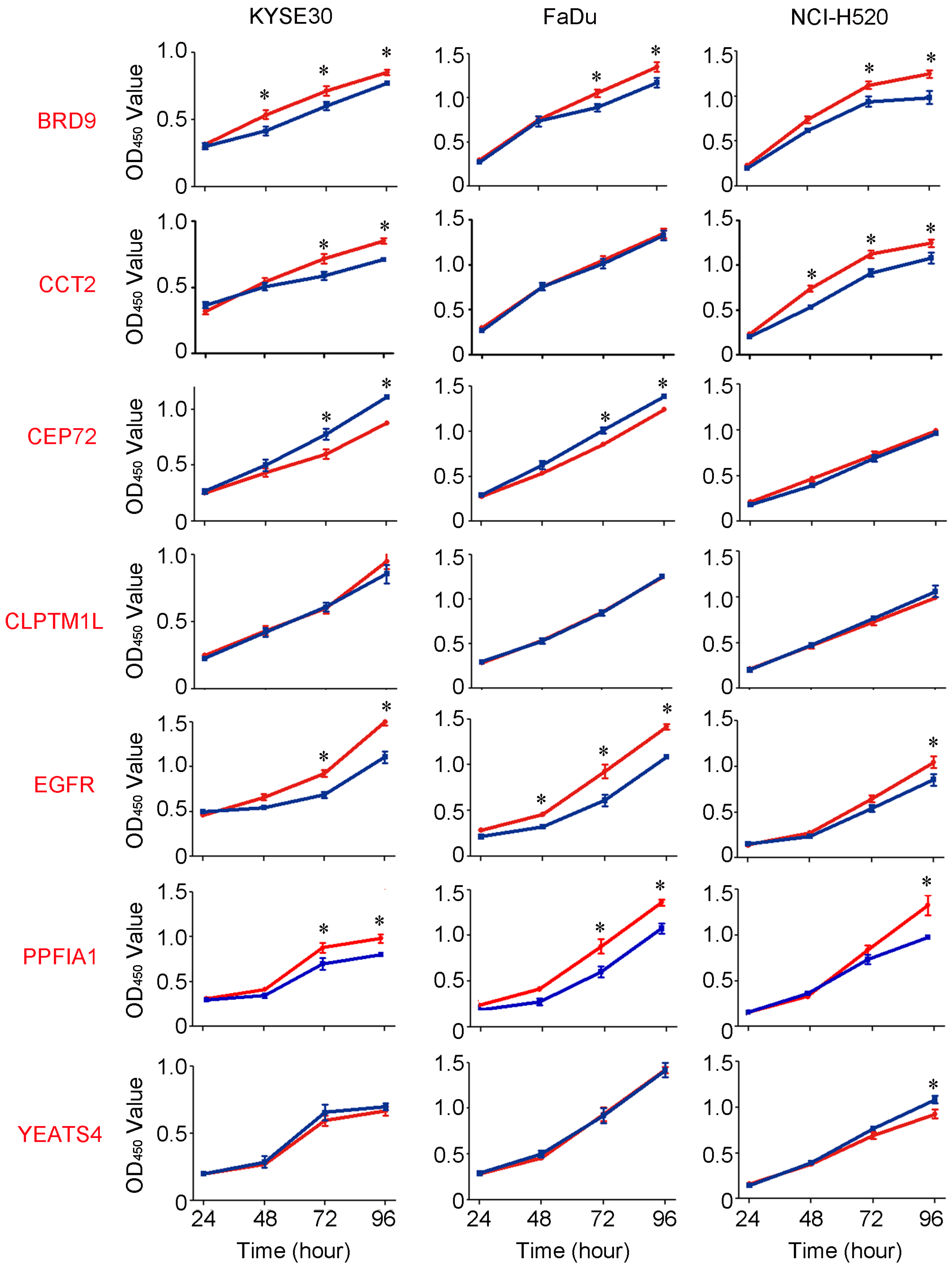

Supplementary Figure 19. Effects of knockdown of 7 genes with copy number gain on SCC cell proliferation. Line in blue represents siRNA and line in red represents scramble RNA. Data represent mean ± s.e.m. of OD_{450} values from three independent experiments and each had triplication. *, *P*<0.01 by Student's *t*-test compared with scramble RNA.

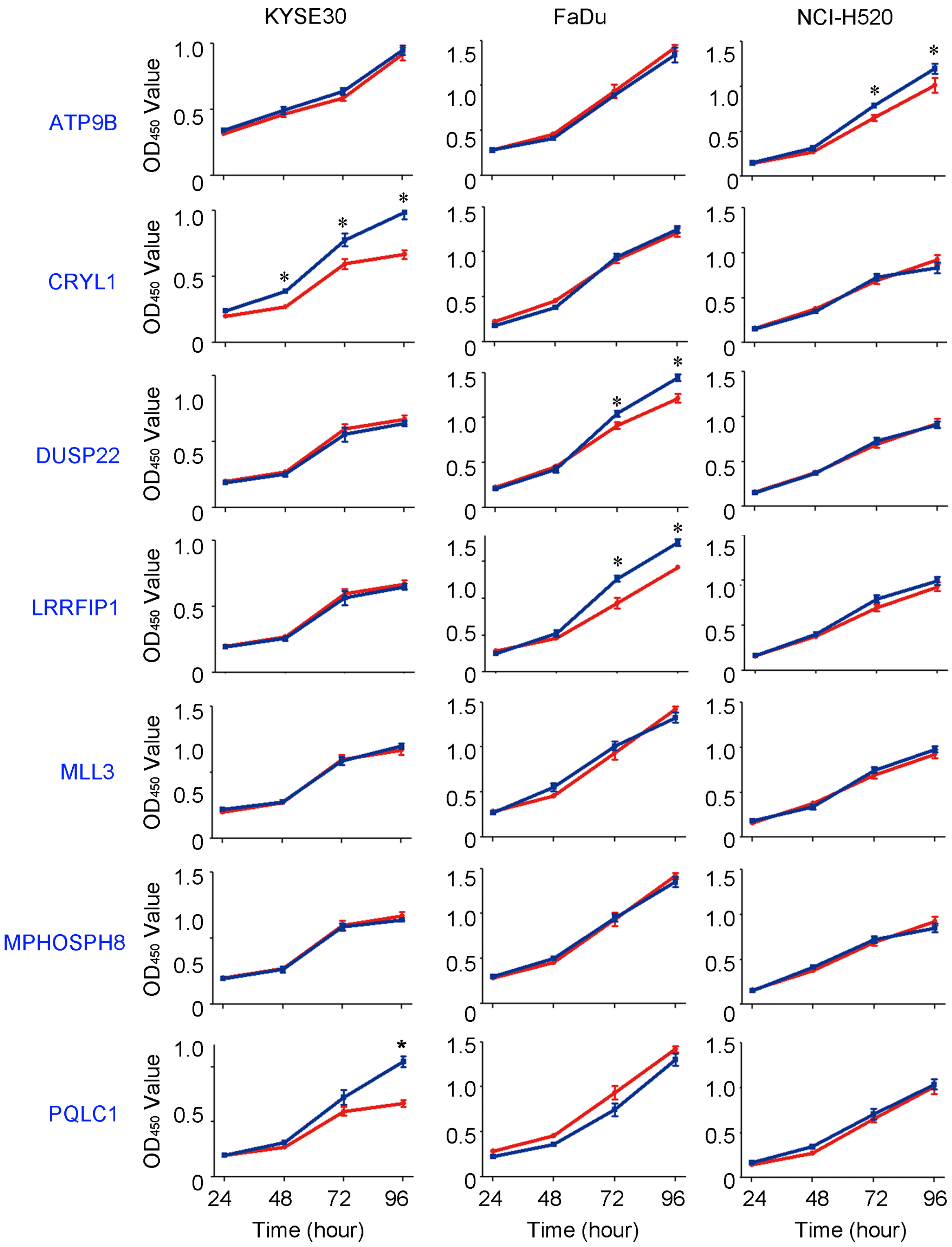

Supplementary Figure 20. Effects of knockdown of 7 genes with copy number loss on SCC cell proliferation. Line in blue represents siRNA and line in red represents scramble RNA. Data represent mean ± s.e.m. of OD_{450} values from three independent experiments and each had triplication. Line in blue represents siRNA and line in red represents scramble RNA. *, *P*<0.01 by Student's *t*-test compared with scramble RNA.

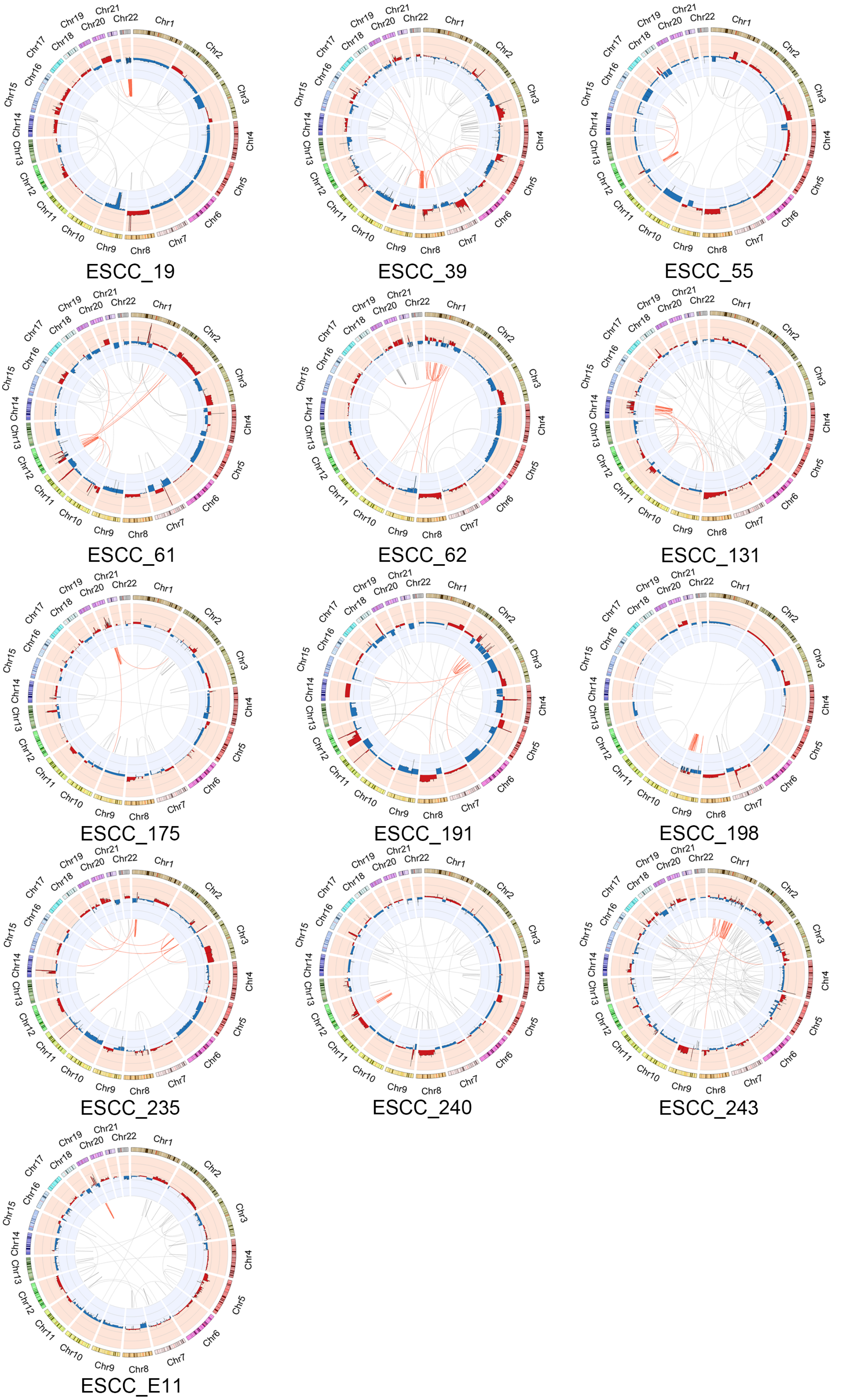

Supplementary Figure 21. Circos plots show chromothripsis in 13 ESCC samples. Chromosomes are shown with different colors and in a circular form. The inner ring represents the copy number variations (red = gain and blue = loss). Lines traversing the ring connects break points of SVs. Sample with chromothripsis shows an extreme amount of rearrangements on one or a few chromosomes.

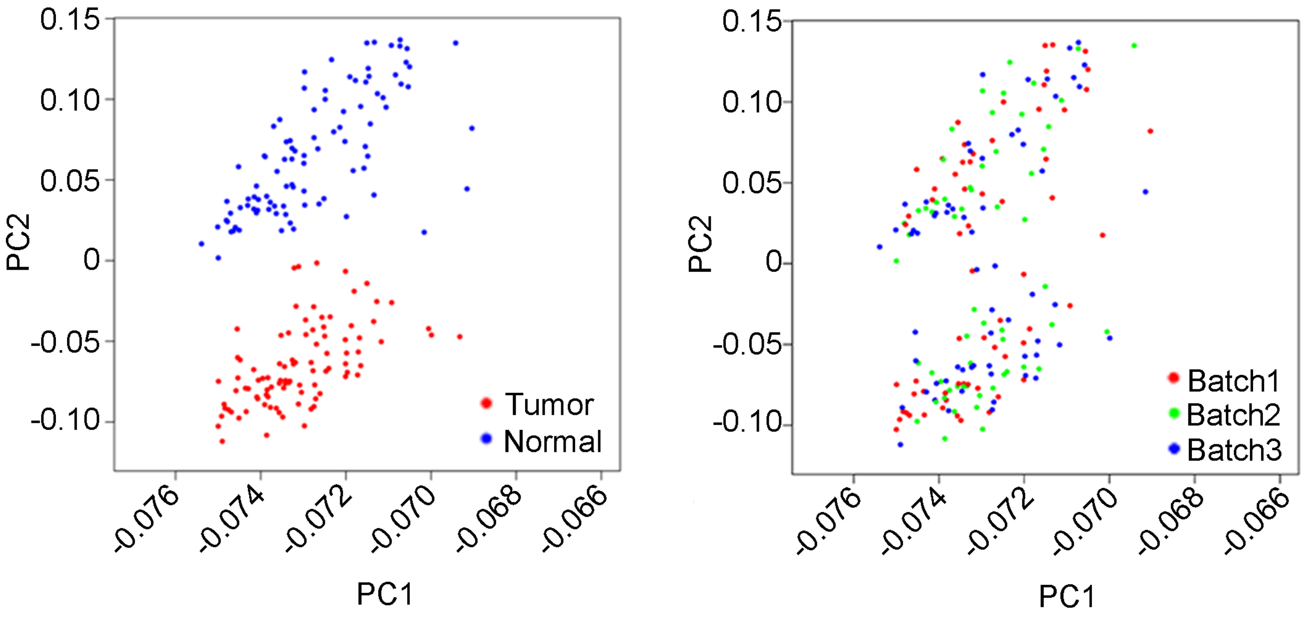

Supplementary Figure 22. Principal component analysis of RNA expression data. Shown are the first two principal components for RNA expression from RNA sequencing data.

Supplementary Table 1. Correlations between mutation or structure variation and clinical phenotype

	P value*		
	**SNV**	**Indel**	**SV**
Age	0.3702	0.2227	0.6573
Gender	0.5249	0.3483	0.0248
Smoking status	0.4916	0.2238	0.6212
Drinking status	0.2863	0.4998	0.4825
TP53 mutation	0.0001	0.0798	0.8253

*Student's *t*-test.

Supplementary Table 2. Kataegis events identified in ESCC samples

Sample ID	Chromosome	Arm	Start	End	Number of mutation	Nearby SV
ESCC_12	11	q	93729585	93731670	8	
ESCC_131	Y	p	8359558	8436129	8	translocation
ESCC_142	3	q	172859671	172861401	8	
ESCC_16	1	p	18566790	18569466	6	
ESCC_16	5	q	95340931	95343787	9	
ESCC_169	5	q	55606291	55610731	8	inversion
ESCC_179	15	q	100989354	100991568	6	
ESCC_191	1	q	226196010	226197541	6	
ESCC_191	6	p	31645348	31647289	7	
ESCC_191	18	q	23029028	23029990	11	translocation
ESCC_208	2	q	119478549	119484512	13	
ESCC_208	6	p	7507845	7523858	22	
ESCC_208	10	q	62385187	62389337	7	
ESCC_E3	9	p	36380923	36386866	8	
ESCC_E3	13	q	23148278	23168312	20	translocation

Supplementary Table 3. Similarity between the mutational signatures in this study and in COSMIC

ESCC (this study)	COSMIC (Alexandrov *et al.*)	Cosine similarity	Correlation with
WES Signature E1	Signature 2	0.819	APOBEC
	Signature 13	0.813	APOBEC
WES Signature E2	Signature 4	0.860	Smoking
WES Signature E3	Signature 1A	0.829	Age
	Signature 1B	0.783	Age
	Signature 6	0.939	Defective DNA mismatch repair
	Signature 15	0.862	Defective DNA mismatch repair
WES Signature E4	Signature 16	0.873	Unknown
WES Signature E5	Signature 1A	0.838	Age
	Signature 1B	0.892	Age
	Signature 6	0.915	Defective DNA mismatch repair
	Signature 15	0.826	Defective DNA mismatch repair
WES Signature E6	--	--	--

Supplementary Table 4. Somatic mutations in the non-coding regions identified by whole-genome sequencing

Chromosome	Start	End	Region type	Gene	Target region mutation number	Target region length	*P* value*	*q* value#
chr11	65190269	65213011	lincRNA_exon	NEAT1	6	22742	2.71×10^{-28}	5.92×10^{-25}
chr1	73801129	73804560	lincRNA_exon	LINC01360	4	3431	1.46×10^{-18}	8.00×10^{-16}
chr5	59816847	59822245	lincRNA_exon	PART1	4	5398	1.46×10^{-18}	8.00×10^{-16}
chr22	48312477	48322013	lincRNA_exon	CTA-280A3.2	4	9536	1.46×10^{-18}	8.00×10^{-16}
chr1	207096343	207098592	promoter	FCMR	5	2250	4.39×10^{-17}	2.17×10^{-13}
chr18	45335328	45368200	3UTR	SMAD2	7	32873	3.23×10^{-41}	1.00×10^{-37}
chr2	32530693	32541663	3UTR	YIPF4	5	10971	4.65×10^{-29}	4.81×10^{-26}
chr12	133494894	133501961	3UTR	ZNF605	5	7068	4.65×10^{-29}	4.81×10^{-26}
chr1	42642210	42645383	3UTR	FOXJ3	4	3174	4.23×10^{-23}	1.31×10^{-20}
chr2	192550492	192561385	3UTR	NABP1	4	10894	4.23×10^{-23}	1.31×10^{-20}
chr4	56294070	56301584	3UTR	CLOCK	4	7515	4.23×10^{-23}	1.31×10^{-20}
chr4	69176105	69179819	3UTR	YTHDC1	4	3715	4.23×10^{-23}	1.31×10^{-20}
chr7	141170584	141180180	3UTR	TMEM178B	4	9597	4.23×10^{-23}	1.31×10^{-20}
chr18	60052265	60058525	3UTR	TNFRSF11A	4	6261	4.23×10^{-23}	1.31×10^{-20}
chr18	74980856	74989852	3UTR	GALR1	4	8997	4.23×10^{-23}	1.31×10^{-20}

**P* values were calculated by a regional recurrence testing approach.

#FDR (false discovery rate).

Supplementary Table 5. Primers used for validation of gene fusions by PCR-Sanger sequencing

Fusion genes	Fusion sample	Break point	Forward primer (5'→3')	Reverse primer (5'→3')
ERC1-WNK1	ESCC_240	chr12:1299718 and chr12:968952	TATCATTAAGCAAAAAAGCAGTT	CAAGTCTACCACTAACCCCAAA
RAD52-ERC1	ESCC_142	chr12:1052385 and chr12:1326966	ACACACCACACCCATCAGAATAA	AGAAACTTTGAGTGGAGGCGAA
PRAF2-ERC1	ESCC_220	chrX:48931714 and chr12:1464097	TTGGTTTGGTAGTAGAGGAGGTTG	TTCTATGGCTACGCTGGTGCT
NRG1-ZCCHC7	ESCC_156	chr8:31523079 and chr9:37341603	ACCAGTTTGCCTTTATGACCTTC	TCTCCTACTACTTTCCCTCACAGC
WT1-MRPL19	ESCC_243	chr11:32414334 and chr2:75884322	CCAGCAATGAGAAGTGAACCTA	CAGCAAATAATCTAAACAAGTGAAG

Supplementary Table 6. Distribution of select characteristics of individuals with ESCC

	No. (%)
**Gender**	
Male	83 (88.3)
Female	11 (11.7)
**Age**	
< 60	42 (44.7)
≥ 60	52 (55.3)
**Smoking status**	
Smoker	77 (81.9)
Non-smoker	17 (18.1)
**Drinking status**	
Drinker	74 (78.7)
Non-drinker	20 (21.3)
**Tumor location***	
Upper	0
Middle	31 (33.0)
Lower	26 (27.7)
Middle and Lower	37 (39.3)
**Stage**	
I	0
II	26 (27.6)
III	67 (71.3)
IV	1 (1.1)
**T stage#**	
T1	0
T2	13 (13.8)
T3	42 (44.7)
T4	39 (41.5)
**N stage#**	
N0	30 (31.9)
N1	26 (27.7)
N2	25 (26.6)
N3	13 (13.8)
**M stage#**	
M0	93 (98.9)
M1	1 (1.1)
**Survival status**	
Deceased	62 (66.0)
Alive	32 (34.0)

*Tumor locations were classified into three regions by the distance from the incisor to the tumor. Upper, 20-25 cm; middle, 25-30 cm and lower, 30-40 cm.

#Tumor TNM stages were determined according to the 7th edition of AJCC TNM staging system of ESCC.

Supplementary Table 7. Primers used for quantitative real-time PCR

Gene ID	Symbol	Forward primer (5'→3')	Reverse primer (5'→3')
8452	CUL3	TCGACAGCTCACACTCCAGCAT	GTGCTTCCGTGTATTAGAGCCAG
3516	RBPJ	TCATGCCAGTTCACAGCAGTGG	TGGATGTAGCCATCTCGGACTG
374868	ATP9B	GAGAATCGCACCTACCAGGCTT	GAATGCAGAAGCTGAGGACCTG
65980	BRD9	GCGACTTGAAGTCGGACGAGAT	GTCCACCACTTTCTTGCTGTAGC
10576	CCT2	GCTCACAGTGAAGGCAATACCAC	GCACTCAGAAGAACCTGTCGCT
55722	CEP72	GGCGAGATTGTGGAACTGAAGC	GCAGGTGTTCATTGGTGCTGAC
81037	CLPTM1L	GGAAAACCGTGCATTACCTGCC	CAGTGAGACCTTGTCGTAGGAC
51084	CRYL1	TGCTGTTTGCCAGTGGAGGCTT	GAGCCTTTCAGAGAACCTGCCT
56940	DUSP22	TGACCGTCACTGACTTTGGCTG	CTTCCTTCAGCCACTGCCGATA
1956	EGFR	CAGATGGATGTGAACCCCGA	CGTAGCATTTATGGAGAGTGAGTC
9208	LRRFIP1	GAGAGACTTCCGACACCCTCAA	CACCTCCACTTCACTGGCTCTT
58508	MLL3	AGATCAGCGTGGACCCTATCCT	CTCTTGACTCGGCATGGTACCA
54737	MPHOSPH8	CTCCTCATCACAAAAGGCGCGA	CAGTCTCACCATTGCTTTGCTGG
8500	PPFIA1	AGCCATGATGCTTCAGGAGCAG	GACTTCCACTGCCAACTCGACT
80148	PQLC1	ACCTACCTGTCCATTGACTCCG	GGTCCACATGAGCACCATCTTG
8089	YEATS4	GCTGTTTCAATCAGACACCAATGC	GGCTCCTAATGTTAGCTGACGAG
2597	GAPDH	TTGGCCAGGGGTGCTAAG	AGCCAAAAGGGTCATCATCTC
